# MicroRNAs in seminal plasma are able to discern infertile men at increased risk of developing testicular cancer

**DOI:** 10.1002/1878-0261.13784

**Published:** 2024-12-16

**Authors:** Carmen Ferrara, Rosalia Battaglia, Angela Caponnetto, Anna Fazzio, Michele Stella, Cristina Barbagallo, Nicolò Musso, Federica Lunelio, Maria Elena Vento, Placido Borzì, Paolo Scollo, Davide Barbagallo, Marco Ragusa, Salvatore Pernagallo, Cinzia Di Pietro

**Affiliations:** ^1^ Section of Biology and Genetics “G. Sichel”, Department of Biomedical and Biotechnological Sciences University of Catania Italy; ^2^ Department of Medicine and Surgery University of Enna "Kore" Enna Italy; ^3^ IVF Unit Cannizzaro Hospital Catania Italy; ^4^ Obstetrics and Gynecology Division, Maternal and Child Department, Cannizzaro Hospital Catania Kore University of Enna Italy; ^5^ DESTINA Genomica S.L. Granada Spain

**Keywords:** circulating microRNA, liquid biopsy, male infertility, testicular germ cell tumor

## Abstract

Male infertility is a risk factor for the development of testicular germ cell tumors. In this study, we investigated microRNA profiles in seminal plasma to identify potential noninvasive biomarkers able to discriminate the men at highest risk of developing cancer among the infertile population. We compared the microRNA profiles of individuals affected by testicular germ cell tumors and healthy individuals with normal or impaired spermiograms using high‐throughput technology and confirmed the results by single‐assay digital PCR. We found that miR‐221‐3p and miR‐222‐3p were downregulated and miR‐126‐3p was upregulated in cancer patients compared to both infertile and fertile men. ROC curve analysis confirmed that miR‐126 upregulation is able to identify cancer patients among the infertile male population. In addition, in‐depth bioinformatics analysis based on weighted gene co‐expression networks showed that the identified miRNAs regulate cellular pathways involved in cancer.

## Graphical abstract

Male infertility is a known risk factor for the development of testicular cancer. In this paper, we analyzed the expression profile of a microRNA panel by real‐time PCR and validated the results by digital PCR. Experimental and bioinformatics analyses allowed us to identify possible biomarkers able to discern men with a higher risk of developing testicular cancer.

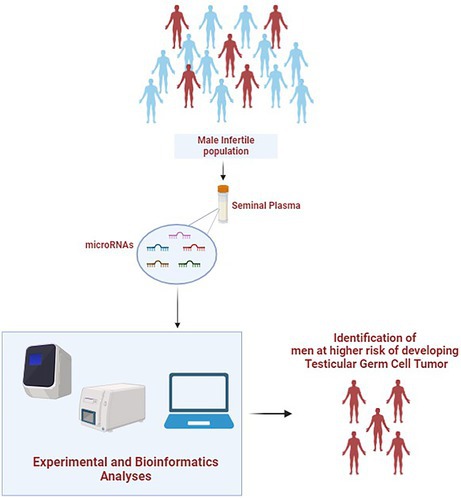

**Abbreviations**
AFP: alpha‐fetoprotein
ANOVA: analysis of variance
AUC: area under the curve
CTRL IS: healthy subjects with impaired spermiogram
CTRL NS: healthy subjects with normal spermiogram
CTRL: healthy subjects
CXCL12: C‐X‐C motif chemokine 12
CXCR4: C‐X‐C motif chemokine receptor 4
DE: differentially expressed
EGFR: epidermal growth factor receptor
FOXO3: forkhead box O3
GEO: Gene Expression Omnibus
GRB10: growth factor receptor bound protein 10
GS: gene significance
hCG: human chorionic gonadotropin
IGFBP2: insulin like growth factor binding protein 2
IVF: *in vitro* fertilization
KEGG: Kyoto Encyclopedia of Genes and Genomes
MBP2: myelin basic protein 2
MIENTURNET: microRNA enrichment turned network
miRNA: microRNA
MM: module membership
NOA: non‐obstructive azoospermia
PGCs: primordial germ cells
PGR: progesterone receptor
ROC: receiver operating characteristic
SOCS3: suppressor of cytokine signaling 3
TBK1: TANK‐binding kinase 1
TGCT: testicular germ cell tumor
TICAM1: Toll‐interleukin 1 receptor domain‐containing adaptor molecule 1
TOM: topological overlap matrix
TOM1: target of myb1 membrane trafficking protein
WHO: World Health Organization

## 1 Introduction

Testicular germ cell tumors (TGCTs) are the most common solid malignancy in young adult men, within the age range of 15–45 years, and their incidence has increased worldwide over the last 20 years. Among several potential risk factors, such as cryptorchidism [1], exposure to endocrine‐disrupting chemicals [2], and genetic abnormalities [3], infertility has attracted the interest of the scientific community [4].

It is known that male fertility can be affected by cancers of the reproductive system, such as testicular and prostate cancer. However, ongoing studies aim to investigate whether male reproductive issues might precede reproductive cancers, to provide a thorough understanding of the intricate links between reproductive dysfunction and male reproductive cancers. Several studies have shown an increased likelihood of TGCTs occurrence in subfertile men [5]. Furthermore, it has been observed that this increased risk persists for up to 11 years after evaluation, suggesting that infertility may be an early marker for TGCTs [6]. While the exact connection between testicular germ cell cancer and infertility remains unclear, existing data strongly support an association between these two conditions [7].

Thanks to *in vitro* fertilization (IVF) techniques, it is possible to obtain viable embryos even in the presence of poor semen quality, by selecting a few morphologically normal spermatozoa. For this reason, male infertility is often neglected and bypassed without further investigations or careful urological examination [8]. This could lead to a delay in the diagnosis of an underlying condition, resulting in a higher risk of progression of the pathology.

Conventional serum biomarkers such as alpha‐fetoprotein (AFP) and human chorionic gonadotropin (hCG) could be used to guide diagnosis and follow‐up of TGCTs, however, they lack sensitivity and specificity [9]. One of the main limitations of the use of AFP is that its serum levels can be altered by clinical conditions other than TGCTs [10]. Similarly, elevated hCG levels, which are primarily produced by components of choriocarcinoma, can also occur in other malignancies [11]. Therefore, there is a need to identify potencial new biomarkers for the early diagnosis of TGCTs.

In recent decades, numerous studies have shown that microRNAs (miRNAs) are significantly dysregulated in human cancers, highlighting their involvement in tumor initiation, progression, and spread [12, 13]. Depending on their targets, miRNAs can act as tumor suppressors by silencing oncogenes or as oncogenes by silencing tumor suppressor genes [14]. miRNAs play a crucial role also in the regulation of spermatogenesis, and their dysregulation has negative effects on male fertility [15]. Deletion of the Dicer gene (Dicer1) in mouse epididymal cells affects epididymal epithelial differentiation, lipid synthesis and sperm maturation [16]. In Sertoli cells, it profoundly affects the testicular proteome, leading to complete absence of spermatozoa and progressive testicular degeneration [17]. Deletion in male germ cells impaired haploid spermatid differentiation, resulting in apoptosis, spermatogenic failure during meiosis and haploid phases [18]. In addition, in primordial germ cells (PGCs), it affected their proliferation and early‐stage spermatogenesis. The cumulative effects of these defects often lead to male infertility.

Several studies have reported the up‐ or downregulation of different miRNAs in men with conditions such as asthenozoospermia, oligoasthenozoospermia, nonobstructive azoospermia (NOA), and teratozoospermia compared to normozoospermic men, suggesting the potential utility of these miRNAs in the diagnosis and treatment of male subfertility/infertility [19].

In addition to the cellular microenvironment, which includes Sertoli cells, Leydig cells, spermatogonia, and mature spermatozoa, miRNAs can also be found in seminal plasma, referred to as circulating or extracellular miRNAs. They may play a role in somatic‐germ line communication, and therefore we believe that their deregulation, due to pathological conditions may be reflected in seminal plasma, making them good biomarkers applicable to liquid biopsy, allowing early, non‐invasive diagnosis.

Several studies have shown a differential expression of extracellular miRNAs in the seminal plasma of infertile men compared to their fertile counterparts [20]. These studies have highlighted the important role of various miRNAs in spermatogenesis, which have been found to be involved in regulating germ cell function, determining cell fate, maintaining undifferentiated stem cell populations, and facilitating cell differentiation throughout spermatogenesis. In this study, we analyzed the expression profile of miRNAs in seminal plasma of men affected by TGCTs and healthy fertile and subfertile subjects in order to identify biomarkers for testicular cancer, considering infertility as one of the main risk factors associated with this pathology. In addition, we performed pathway and gene co‐expression network analysis to elucidate the biological role of the identified miRNAs and their target genes in testicular cancer.

## 2 Materials and methods

### 2.1 Samples collection

Seminal plasma samples were collected from TGCTs patients who were undergoing sperm cryopreservation prior to chemotherapy and from healthy patients with both impaired or normal spermiograms undergoing assisted fertilization procedures at the IVF Center in Cannizzaro Hospital, Catania, Italy. Specifically, we conducted an initial analysis on 9 samples, 3 for each group, and then a validation of the differentially expressed (DE) miRNAs on 24 samples, 8 for each group, for two miRNAs, and on 18 samples, 6 for each group, for one miRNA. The samples were collected from January 2021 to January 2024.

Although seminal plasma is classified as waste material, this study was reviewed and approved by the Ethics Committee of Kore University of Enna (Protocol Number 26578) and conducted in full compliance with ethical guidelines, with the understanding and written informed consent obtained from all participants.

Our research followed the tenets of the Declaration of Helsinki. Semen samples were produced by masturbation after at least 24 h of sexual abstinence and collected in sterile sample containers. The sperm samples were placed for 30 min at 37 °C and then seminal plasma was purified using density‐gradient centrifugation and stored at −80 °C prior to use. The samples were divided into three experimental groups: TGCT patients (TGCT), healthy subjects with normal spermiogram (CTRL NS), and healthy subjects with impaired spermiogram (CTRL IS) according to the WHO guidelines on seminal fluid evaluation 2021. A more detailed description of semen parameters in the three analyzed groups is reported in Tables 1 and 2.

**Table 1:** Description of semen parameters in the three analyzed groups. CTRL IS, healthy subjects with impaired spermiogram; CTRL NS, healthy subjects with normal spermiogram; TGCT, patients affected by testicular germ cell tumors.

Group	Parameter	Mean ± SEM	Minimum	Maximum
TGCT	Total sperm count	41.06 ± 16.4	0.8	108
	% Sperm motility	38.63 ± 4.22	20	57
	% Abnormal morfology	97 ± 0.46	95	99
	Age	28.13 ± 1.34	20	32
CTRL IS	Total sperm count	58.27 ± 30.25	0	212
	% Sperm motility	23.13 ± 6.33	0	46
	% Abnormal morfology	98.13 ± 0.44	97	100
	Age	40.25 ± 3.06	29	54
CTRL NS	Total sperm count	178.9 ± 34.83	82.5	375
	% Sperm motility	61.88 ± 4.29	45	83
	% Abnormal morfology	94.880 ± 0.35	93	96
	Age	36.88 ± 1.54	30	41

**Table 2:** Comparison of the sperm parameters between the three groups. Description of semen parameters in the three analyzed groups. CTRL IS, healthy subjects with impaired spermiogram; CTRL NS, healthy subjects with normal spermiogram; TGCT, patients affected by testicular germ cell tumors. Statistical analysis was performed by one‐way analysis of variance (ANOVA) applying Tukey's method for multiple comparisons. *p‐value (0.05).

Comparison	Parameter	Adjusted *P*‐value
TGCT vs. CTRL IS	Total sperm count	0.9034
	% Sperm motility	0.0993
	% Abnormal morfology	0.1662
TGCT vs. CTRL NS	Total sperm count	0.0065*
	% Sperm motility	0.01*
	% Abnormal morfology	0.0049*
CTRL IS vs. CTRL NS	Total sperm count	0.0173*
	% Sperm motility	< 0.0001*
	% Abnormal morfology	< 0.0001*

### 2.2 RNA isolation, miRNA profiling, and validation

RNA was isolated and purified from 400 μL of seminal plasma using Qiagen miRNeasy Serum/Plasma Kit (Qiagen, GmbH, Hilden, Germany), according to manufacturer's instructions for purification of total RNA, including small RNAs, from serum or plasma. Subsequently, RNA concentration and quality were determined using a NanoDrop One/OneC Microvolume UV–Vis Spectrophotometer.

A discovery analysis was conducted on nine samples (3 for each group) using Qiagen miRCURY LNA miRNA serum/plasma focus PCR panel. Since no specific panel is available for seminal plasma, we chose to use this one, which contains 88 miRNAs enriched in serum and plasma, among which there are some of the main miRNAs whose deregulation is related to both TGCTs and/or infertility, enriched in serum and/or plasma [21, 22]. The list of miRNAs analyzed in the miRCURY LNA miRNA serum/plasma focus PCR panel is reported in Table S1. Total RNA was reverse transcribed using miRCURY LNA RT Kit (Qiagen), in a final volume of 20 μL. According to the manufacturer's instructions, the reaction solution was subjected to a thermal cycle of 42 °C for 60 min, 95 °C for 5 min, and hold at 4 °C. The entire reaction volume was then combined with the miRCURY LNA miRNA serum/plasma focus PCR panel reaction mix, containing the miRCURY SYBR Green Master Mix, ROX Reference Dye, and RNAse‐free water, for a final volume of 1 mL that was divided into 10 μL per well. Quantitative RT‐PCR reactions were performed on a QuantStudio 7 Flex Real‐Time PCR System (Applied Biosystem, Life Technologies, Waltham, MA, USA) as follows: 95 °C for 2 min, followed by 40 amplification cycles of 95 °C for 10 s and 56 °C for 1 min.

Validation of the DE miRNAs, resulting from the expression profiling analysis, was performed by QIAcuity digital PCR on 24 samples (8 for each group) for miR‐221‐3p and miR‐222‐3p and on 18 samples (6 for each group) for miR‐126‐3p.

cDNA was produced using TaqMan MicroRNA Reverse Transcription Kit (Thermo Fisher Scientific, Waltham, MA, USA) using primers for miR‐specific RT (Thermo Fisher Scientific). Digital PCR was performed on a QIAcuity One Digital PCR System by using TaqMan miRNA probes (Thermo Fisher Scientific, Assays ID: 002228, 000524, 002276) and setting a thermal cycle as follows: 95 °C for 2 min, followed by 40 amplification cycles of 95 °C for 15 s and 60 °C for 30 s. Data normalization was performed using the miR‐29a‐3p gene as endogenous control, as it resulted to be the most stable miRNA in the miRCURY panel. Statistical analysis was performed by one‐way analysis of variance (ANOVA) applying Tukey's method for multiple comparisons, using graphpad prism v8.4.2 (GraphPad Software, Boston, MA, USA); *P*‐values < 0.05 were considered statistically significant.

### 2.3 Receiver operator characteristic curve analysis and correlation

Ratio copies per μL values of the DE miRNAs were used to perform a receiver operator characteristic (ROC) curve analysis using medcalc statistical software v19.2.6 (MedCalc Software Ltd, Ostend, Belgium).

The correlation between ratio copies per μL values from digital PCR analysis and sperm parameters was calculated using Pearson's correlation method, and statistical significance was calculated using a two‐tailed unpaired *t*‐test, using graphpad prism v8.4.2; only *r*‐values ≥ 7 and *P*‐values < 0.05 were considered statistically significant.

### 2.4 Computational analysis

To investigate the biological role of the DE miRNAs, their experimentally validated targets were retrieved from miRTarBase 9.0 (https://mirtarbase.cuhk.edu.cn/~miRTarBase/miRTarBase_2019/php/download.php) [23]. The network of miRNA–mRNA interactions was constructed and analyzed by cytoscape v3.10.1 (Cytoscape Consortium, University of California, San Diego, CA, USA). The nodes were ranked according to the betwenness centrality and shown with a scale color from a higher score (dark blue) to a lower score (lilac).

We considered the betweenness as a measure of centrality instead of the degree. In fact, the betweenness is able to highlight the node importance also in the cases with low degree, since it represents the ratio of the shortest paths passing through a node among all of the shortest paths in the network.

Kyoto Encyclopedia of Genes and Genomes (KEGG) analysis was performed, and the individuated pathways and their relative *P*‐values were obtained from MicroRNA ENrichment TURned NETwork (MIENTURNET) (http://userver.bio.uniroma1.it/apps/mienturnet/) [24]. Only pathways with *P*‐values ≤ 0.05 were considered.

### 2.5 Gene co‐expression network analysis

In order to prioritize miRNA targets in our study, a gene co‐expression network analysis was performed. Gene expression profiles and clinical traits of GSE1818 were downloaded from the GEO database (https://www.ncbi.nlm.nih.gov/geo/query/acc.cgi). For this analysis, only the three normal testis and all the tumoral tissues (5 embryonal carcinoma, 1 choriocarcinoma, 4 teratoma, 3 seminoma, and 4 Yolk sac tumor) from this dataset were used. We built an adjacency matrix, for an unsigned network, applying Pearson's correlation method, describing the correlation strength between the nodes (genes), using a soft‐threshold β = 14 with a scale‐free *R* ^2^ = 0.89. Then, we transformed the adjacency matrix into a topological overlap matrix (TOM) and identified modules by hierarchical clustering, using 30 as the minimum module size. We merged similar modules with an abline of 0.25. Once the modules were identified, we performed a correlation analysis between them and the characteristics of our samples (tumor or nontumor). We identified the modules most closely related to the trait of our interest and extrapolated the corresponding genes. Among these genes, we identified the experimentally validated targets of the DE miRNAs, obtained from miRTarBase (https://mirtarbase.cuhk.edu.cn/~miRTarBase/miRTarBase_2019/php/download.php), and constructed a weighted network for each module by cytoscape. Using the same dataset, we performed a differential expression study by the limma r package, applying the Bonferroni method to calculate adjusted *P*‐values. The code of the analyses is available on GitHub at https://github.com/TGCTmiRNA.

## 3 Results

### 3.1 miRNA profiling and validation

**Fig. 1:**
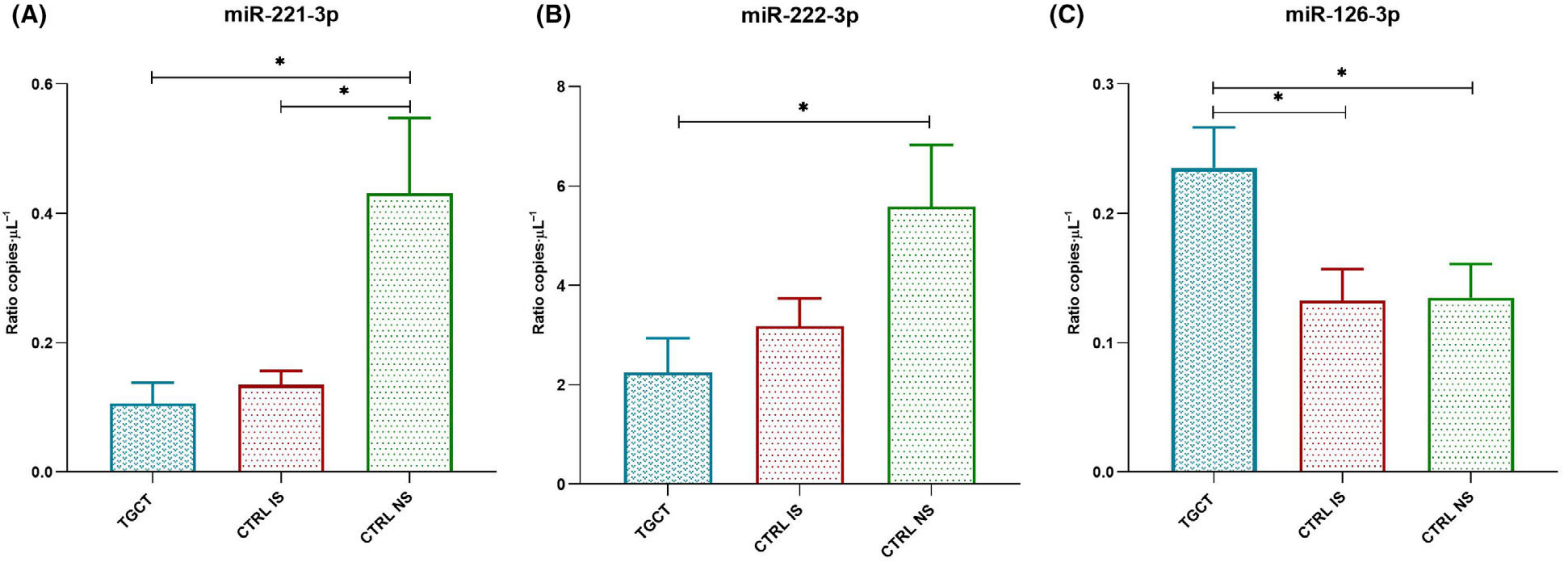
Bar plot of differentially expressed miRNAs from Digital PCR analysis. (A) differential expression of miR‐221‐3p (*P < 0.05, evaluated using ANOVA. Error bars indicate SD). (B) differential expression of miR‐222‐3p (*P < 0.05, evaluated using ANOVA. Error bars indicate SD). (C) differential expression of miR‐126‐3p (*P < 0.05, evaluated using ANOVA. Error bars indicate SD). CTRL IS, healthy subjects with impaired spermiogram; CTRL NS, healthy subjects with normal spermiogram; TGCT, patients affected by testicular germ cell tumors. In *Y*‐axis, values are reported as ratio copies·μL^−1^.

The miRCURY LNA miRNA serum/plasma focus PCR panel analysis revealed three DE miRNAs. In particular, miR‐221‐3p and miR‐222‐3p resulted downregulated and miR‐126‐3p upregulated in TGCT patients with respect to controls. More in detail, miR‐221 and miR‐222 were downregulated in TGCTs vs both IS and NS controls, while miR‐126 showed a significant upregulation in TGCTs vs IS controls. The values of fold changes and *P*‐values of all miRNAs in the panel for each comparison are available in Table S2. The expression of the three DE miRNAs was subsequently validated through digital PCR, showing a downregulation of miR‐221‐3p comparing TGCT patients versus CTRL NS and CTRL IS versus CTRL NS (Fig. 1A). It also confirmed the downregulation of miR‐222‐3p (Fig. 1B) in TGCT patients with respect to CTRL NS and the upregulation of miR‐126‐3p in TGCT versus CTRL NS and CTRL IS (Fig. 1C).

### 3.2 ROC curve and correlation analyses

**Fig. 2:**
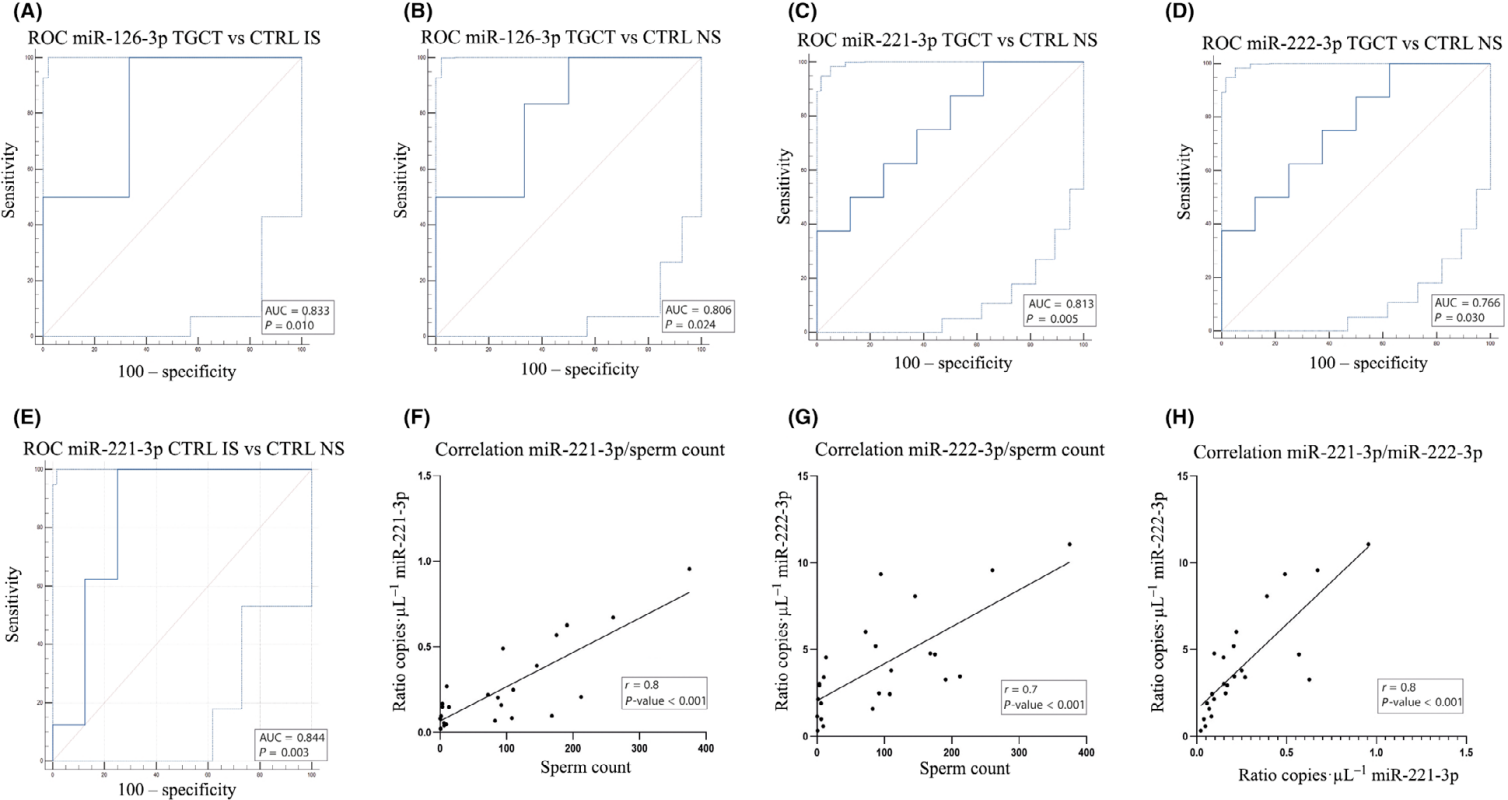
Classical univariate ROC curve analyses and correlation scatterplot. (A) miR‐126‐3p in the comparisons between TGCTs and CTRLs IS. (B) miR‐126‐3p in the comparisons between TGCTs and CTRLs NS. (C) miR‐221‐3p and (D) miR‐222‐3p in the comparisons between TGCTs and CTRLs NS. (E) miR‐221‐3p in the comparisons between CTRLs IS and CTRLs NS. (F) correlation between miR‐221‐3p normalized copies and sperm count. (G) correlation between miR‐222‐3p normalized copies and sperm count. (H) Correlation between miR‐221‐3p and miR‐222‐3p normalized copies. Statistical significance for correlation analysis was calculated using *t*‐test. CTRL IS, healthy subjects with impaired spermiogram; CTRL NS, healthy subjects with normal spermiogram; TGCT = patients affected by testicular germ cell tumors. Area under the ROC curve (AUC) and *P*‐values are shown, *P*‐value < 0.05 were considered statistically significant, the light blue curves in ROC curves indicate the interval of confidence. Statistical significance for ROC curve analysis was calculated using DeLong's method.

The ROC curve analysis revealed that miR‐126‐3p can be used to effectively distinguish TGCT patients from CTRLs IS (AUC = 0.833, *P*‐value = 0.01) (Fig. 2A) and TGCTs from CTRLs NS (AUC = 0.806, *P*‐value = 0.02) (Fig. 2B). Moreover, ROC curve analysis demonstrated that miR‐221‐3p (AUC = 0.813, *P*‐value = 0.005) and miR‐222‐3p (AUC = 0.766, *P*‐value = 0.03) can distinguish TGCTs from CTRLs NS (Fig. 2C,D). In addition, ROC curve analysis for miR‐221‐3p showed an AUC of 0.844 with *P*‐value of 0.003 in distinguish CTRLs IS from CTRLs NS (Fig. 2E). The correlation analysis showed a positive correlation between miR‐221‐3p and miR‐222‐3p and sperm count, with an *r*‐value of 0.8 and 0.7, respectively, and a *P*‐value < 0.0001 (Fig. 2F,G). In addition, a positive correlation has been found also between the expression of the two miRNAs, with an *r*‐value of 0.8 and a *P*‐value < 0.0001 (Fig. 2H).

### 3.3 miRNA‐mRNA network

In order to investigate the biological role of DE miRNAs, we explored the interaction with their targets by constructing an interaction network including all the miRNAs and their respective targets. Our aim was to identify other possible important nodes, i.e., miRNA's targets that may be central in our network. We considered the betweenness as ranking parameter instead of degree since, due to the nature of the data used, miRNAs would certainly have been the most central nodes. The betweenness considers the shortest paths passing through a node, and it can be very efficient to decipher the node importance, especially for those nodes that, despite having few connections and thus a low degree, connect central nodes together [25].

**Fig. 3:**
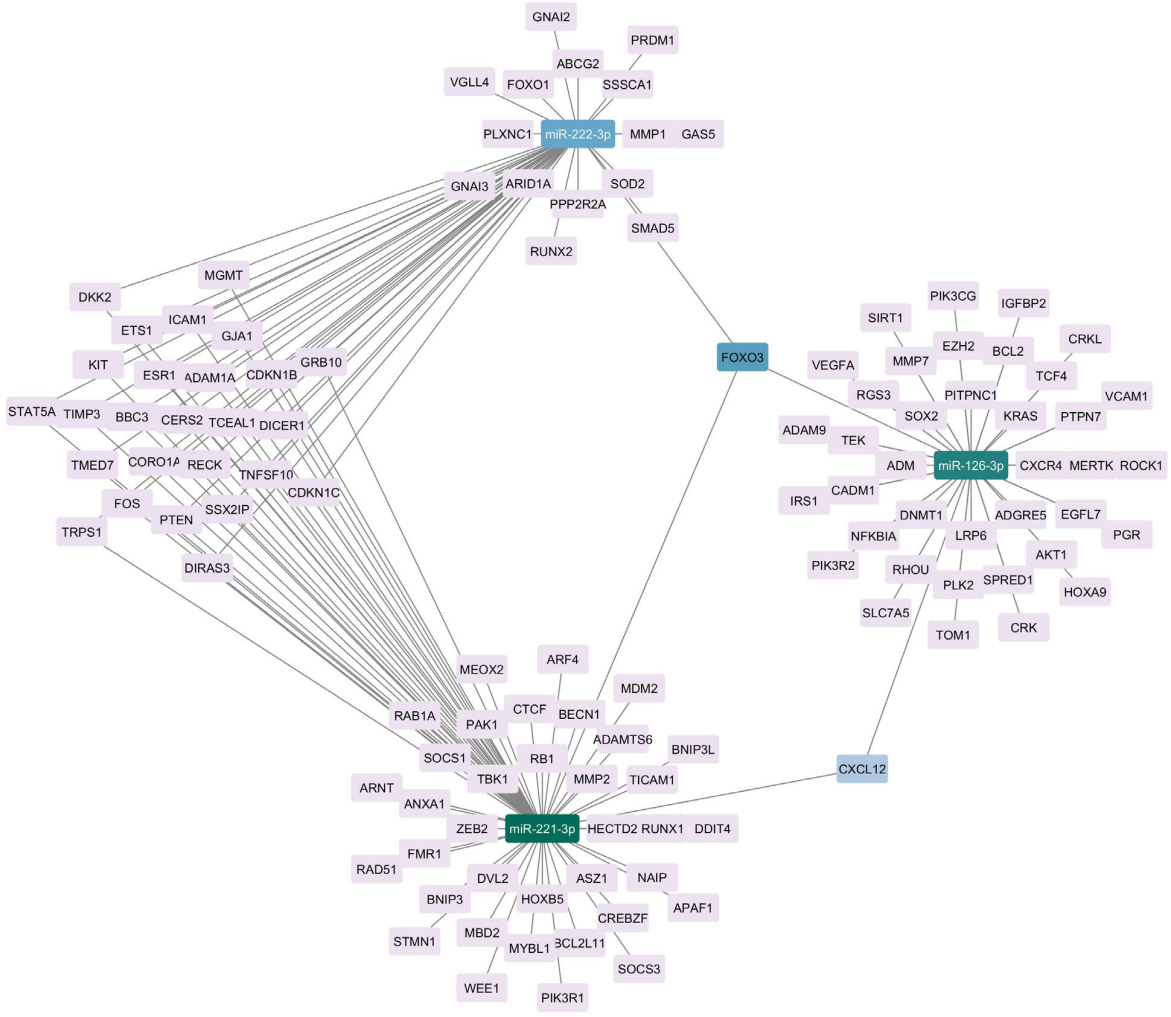
Regulatory network of DE miRNA–mRNA interactions. Nodes are represented with a color scheme according to their betweenness centrality, from dark blue (high centrality) to lilac (low centrality). DE, differentially expressed.

The analysis of the DE miRNA‐target interaction network revealed that miR‐221‐3p (score 0.61), miR‐126‐3p (score 0.51), FOXO3 (score 0.33), miR‐222‐3p (score 0.29), CXCL12 (score 0.13), and miR‐197‐3p (score 0.12) are the nodes with the higher score, based on betweenness centrality (Fig. 3).

### 3.4 Gene co‐expression network analysis

In order to identify, among the multiple targets of our miRNAs, those most likely to be directly associated with TGCTs, we conducted a co‐expression network analysis retrieving gene expression profiles and clinical traits of GSE1818 from GEO database (https://www.ncbi.nlm.nih.gov/geo/query/acc.cgi). We chose to use a dataset from tissue samples since we did not dispose of appropriate tissue samples for analysis. This could be an effective way to study the biological role of our DE miRNAs directly in the tissue, where miRNAs exert their action.

After constructing an unsigned network, 34 modules were identified by hierarchical clustering and dynamic tree cut. Since this is an unsigned network, the correlation of the modules with our trait of interest, i.e., testicular germ cell tumor, was studied as an absolute value. Three modules related to the trait in question emerged from this analysis: the cyan module with an *r*‐value of 0.97 and *P*‐value < 0.0001, the dark magenta module with an *r*‐value of 0.84 and *P*‐value < 0.0001, and the orange module with an *r*‐value of 0.7 and a *P*‐value ≤ 0.0005.

**Fig. 4:**
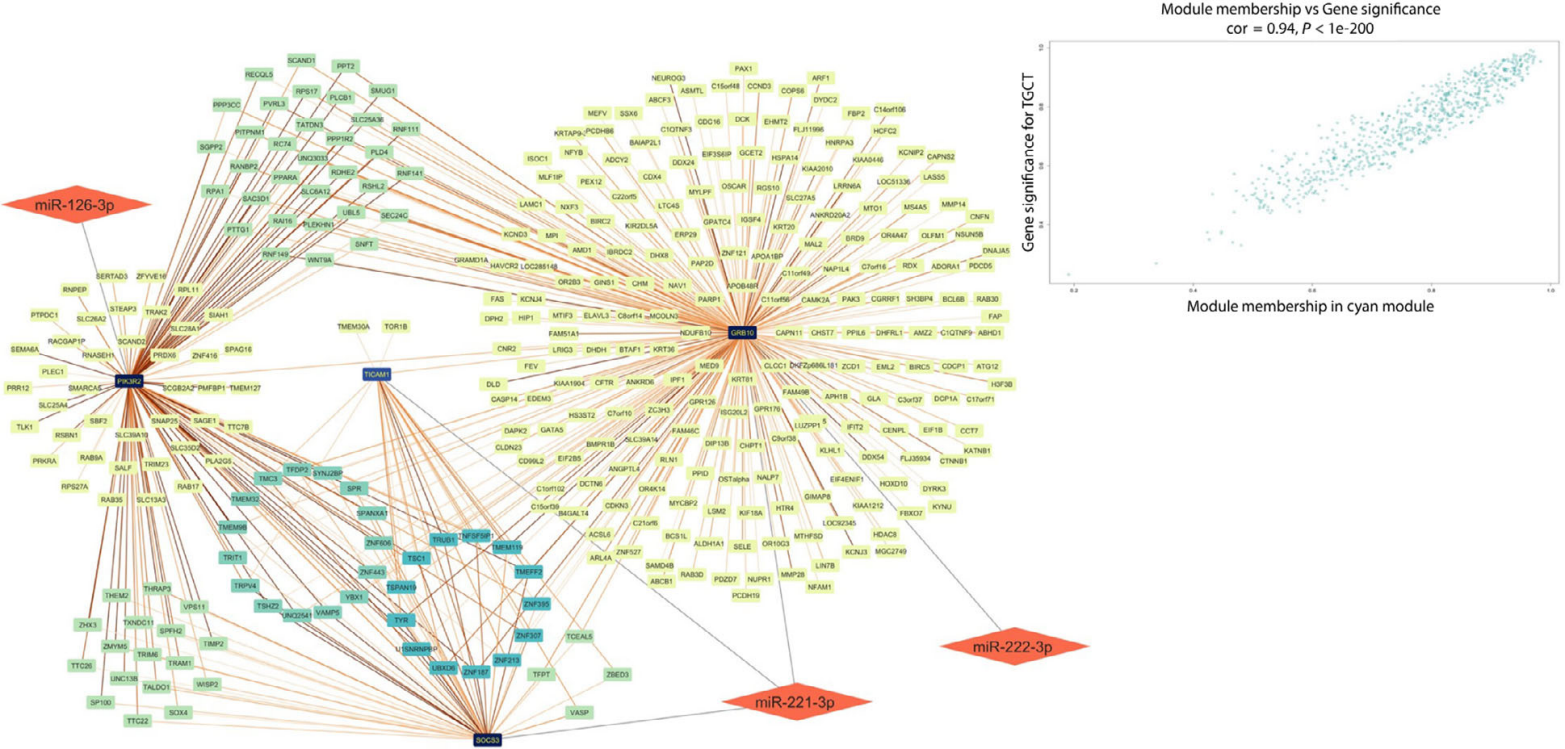
Network of DE miRNAs' target and correlated genes interactions in Cyan module. Nodes are represented with a color scheme according to their degree, from dark blue (high degree) to yellow (low degree). The edges' color indicates the weight of the interaction, with a color scheme from dark red (higher weight) to pale red (lower weight). Rhomboidal nodes contain miRNAs that regulate their respective targets connected to them with an unweighted edge. In the up‐right corner is showed a scatterplot showing MM‐GS correlations for the Cyan module. DE, differentially expressed; GS, gene significance; MM, module membership.

**Fig. 5:**
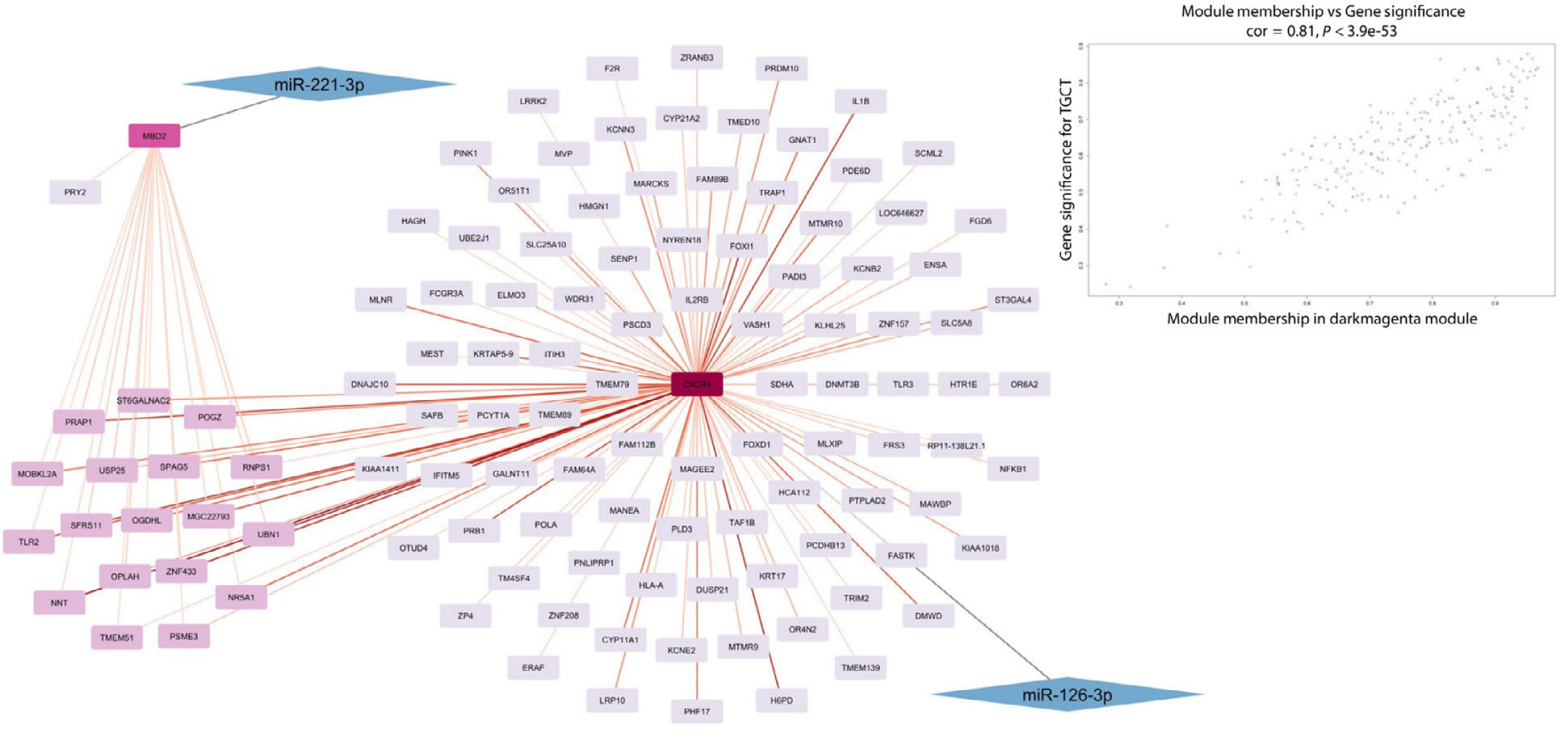
Network of DE miRNAs' target and correlated genes interactions in Dark magenta module. Nodes are represented with a color scheme according to their degree, from dark purple (high degree) to lilac (low degree). The edges' color indicates the weight of the interaction, with a color scheme from dark red (higher weight) to pale red (lower weight). Rhomboidal nodes contain miRNAs that regulate their respective targets connected to them with an unweighted edge. In the upright corner is showed a scatterplot showing MM‐GS correlations for the dark magenta module. DE, differentially expressed; GS, gene significance; MM, module membership.

**Fig. 6:**
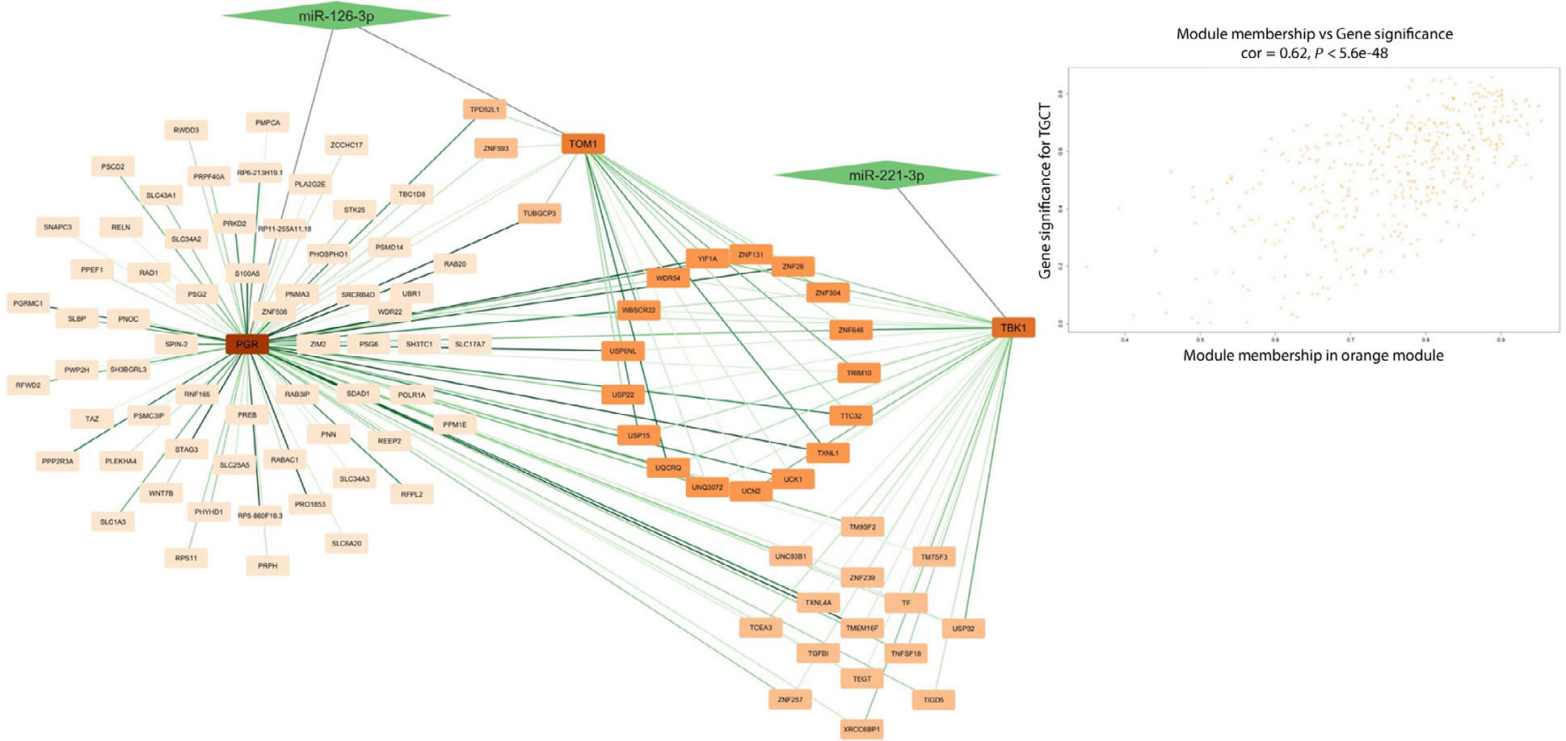
Network of DE miRNAs' target and correlated genes interactions in orange module. Nodes are represented with a color scheme according to their degree, from dark orange (high degree) to pale orange (low degree). The edges' color indicates the weight of the interaction, with a color scheme from dark green (higher weight) to pale green (lower weight). Rhomboidal nodes contain miRNAs that regulate their respective targets connected to them with an unweighted edge. In the up‐right corner is showed a scatterplot showing MM‐GS correlations for the orange module. DE, differentially expressed; GS, gene significance; MM, module membership.

Module membership (MM) and gene significance (GS) were calculated for each gene contained in the three modules. Then, for each module, the correlation between MM and GS was calculated. The cyan module showed a MM‐GS correlation of 0.94 (Fig. 4), the dark magenta module had a MM‐GS correlation of 0.81 (Fig. 5), and the orange module of 0.62, all three presented *P*‐values ≤ 0.0001 (Fig. 6).

The cyan module contains 738 genes of which 4 are targets of 3 of our DE miRNAs, in particular, PIK3R2 (MM = 0.9, GS = 0.89) which is a target of miR‐126‐3p SOCS3 (MM = 0.87, GS = 0.73) and TICAM1 (MM = 0.68, GS = 0.67) which are targets of miR‐221‐3p, and GRB10 (MM = 0.8, GS = 0.83) which is a target of both miR‐221‐3p and miR‐222‐3p. In Fig. 4, we reported as central nodes the 4 targets of our miRNAs belonging to the cyan module and only the genes whose expression is correlated to these targets.

The dark magenta module contains 223 genes of which 2 are targets of 2 of our DE miRNAs: CXCR4 (MM = 0.8, GS = 0.65) which is a target of miR‐126‐3p and MBP2 (MM = 0.6, GS = 0.42) which is a target of miR‐221‐3p. In Fig. 5, we reported as central nodes the two targets of our miRNAs belonging to the dark magenta module and only the genes whose expression is correlated to these targets.

The orange module contains 439 genes of which 3 are targets of 2 of our DE miRNAs: PGR (MM = 0.67, GS = 0.5) and TOM1 (MM = 0.66, GS = 0.33) which are targets of miR‐126‐3p and TBK1 (MM = 0.73, GS = 0.5) which is a target of miR‐221‐3p. In Fig. 6 we reported as central nodes the three targets of our miRNAs belonging to the cyan module and only the genes whose expression is correlated to these targets.

### 3.5 Differential expression analysis of miRNA target genes

**Fig. 7:**
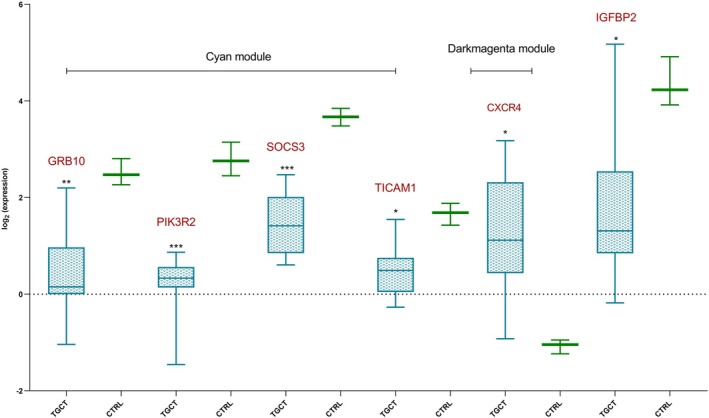
Boxplot of genes differentially expressed in TGCT tissue and belonging to the identified modules. CTRL, healthy subjects; TGCT, patients affected by testicular germ cell tumors. All the samples are derived from the GEO dataset GSE1818. In the *Y* axes, values are shown as log_2_ of normalized expression values. The whiskers represent the highest and lowest values. *P < 0.05, **P < 0.01, ***P < 0.001. Statistical significance was calculated using the moderated *t*‐test. *P*‐values were adjusted for multiple testing using the Benjamini‐Hochberg (BH) method.

Using the same dataset employed for gene co‐expression network analysis (GSE1818), we performed a differential expression study using the limma r package. From the analysis of the same sample set (5 embryonal carcinomas, 1 choriocarcinoma, 4 teratomas, 3 seminomas, and 4 Yolk sac tumors) and gene set, 109 genes resulted to be overexpressed and 962 down‐expressed in TGCTs tissue respect to CTRL tissue. DE genes also include the six of the DE miRNAs targets. Values of fold changes and *P*‐values are available in Table S3. Specifically, the four belonging to the cyan module (GRB10, PIK3R2, SOCS3, and TICAM1) were found to be down‐expressed; CXCR4, belonging to the dark magenta module and target of miR‐126‐3p, been found to be overexpressed; and IGFBP2, not belonging to any of the identified modules but being a target of miR‐126‐3p, was found to be down‐expressed (Fig. 7).

### 3.6 Pathways enrichment analysis

**Fig. 8:**
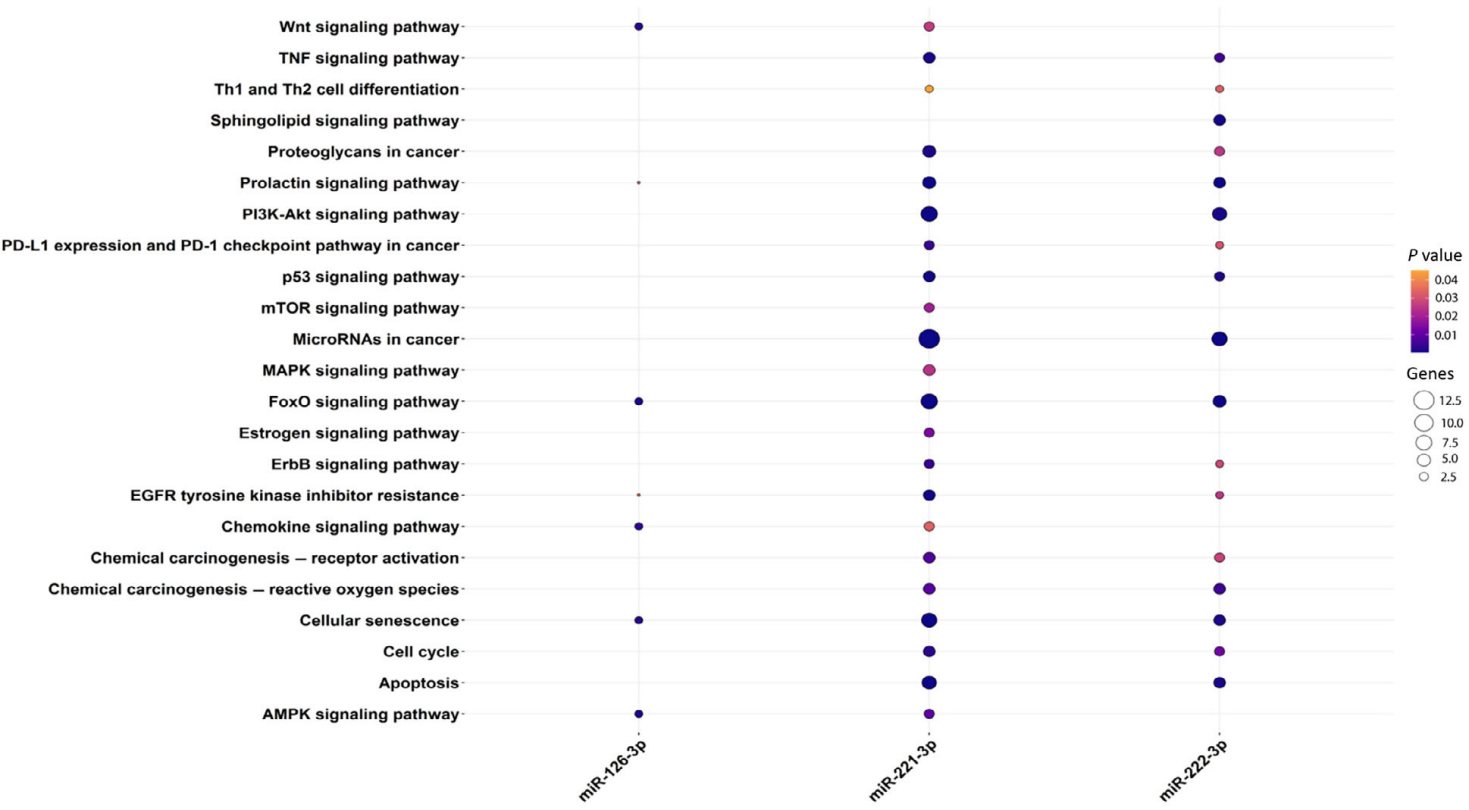
Significant pathways associated with the four DE miRNAs. The point size indicates the number of genes target of DE miRNAs involved in the pathway, while the gradient color indicates the relative *P*‐value. DE, differentially expressed.

We identified four pathways common to all three miRNAs: prolactin signaling pathway, FoxO signaling pathway, EGFR tyrosine kinase inhibitor resistance, and cellular senescence (Fig. 8). Interestingly, two of the previously highlighted miRNAs' target genes are involved in those pathways. In particular, SOCS3 and PIK3R2 are involved in the prolactin signaling pathway and PIK3R2 in FoxO signaling pathway, EGFR tyrosine kinase inhibitor resistance, and cellular senescence.

## 4 Discussion

In recent years, liquid biopsy has received increasing attention in both basic research and precision medicine, in order to develop noninvasive method for diagnosis, prognosis, and therapy in oncology. Among the different biomarkers, circulating miRNAs seem to be the most promising [26].

Regarding TGCTs, miR‐371‐3p may represent a possible serum biomarker [27], but its diagnostic value has not been demonstrated in seminal plasma and, to date, little is known about the miRNA expression profile in seminal plasma of patients with TGCTs.

In this study, we identified miR‐221‐3p, miR‐222‐3p, and miR‐126‐3p as biomarkers in seminal plasma able to discriminate TGCT patients from healthy controls and especially from subfertile men attending an IVF centre (Table 1).

Specifically, we found a statistically significant downregulation of miR‐221‐3p and miR‐222‐3p between TGCT patients and fertile controls and also a significant downregulation of miR‐221‐3p between fertile and subfertile controls (Fig. 1). Furthermore, ROC analysis confirmed that both miRNAs were able to discriminate TGCT patients from fertile men and that miR‐221‐3p discriminated subfertile men from fertile ones (Fig. 2C–E). These data suggest that the downregulation of these miRNAs may be more related to infertility rather than cancer. We also found a positive correlation between the expression levels of the two miRNAs and total sperm count (Fig. 2F,G). Furthermore, consistent with the fact that miR‐221‐3p and miR‐222‐3p belong to the same cluster, we demonstrated a positive correlation between their expression (Fig. 2H).

We also found a significant upregulation of miR‐126‐3p in TGCT patients compared to both fertile and subfertile controls (Fig. 1). ROC analysis for miR‐126‐3p showed that it could discriminate TGCT patients from both fertile and subfertile men, suggesting that this miRNA may be a valid candidate biomarker for testicular cancer (Fig. 2A,B).

The biological function of the three miRNAs, investigated through an in‐depth bioinformatics approach and literature review, confirmed that their altered expression in seminal plasma could explain the alteration of biological pathways involved in infertility and cancer. Indeed, their mRNA targets are enriched in the prolactin signaling pathway, the FoxO signaling pathway, PI3K‐Akt signaling pathway, and cellular senescence (Fig. 8). The gene coexpression network identified modules of co‐expressed genes that could be correlated with TGCTs. Among the targets included in the cyan module are PIK3R2, which is a target of miR‐126‐3p, SOCS3, and TICAM1, which are targets of miR‐221‐3p, and GRB10, which is a target of both miR‐221‐3p and miR‐222‐3p. Among these, PIK3R2, SOCS3, and GRB10 show the highest values of MM and GS, demonstrating that they are the ones with a higher correlation with both the module and the tumor (Fig. 4). Interestingly, the cyan module shows the highest and most significant correlation with the tumor. Furthermore, from the expression analysis performed on the same dataset, these targets were downregulated in tumor tissue compared to normal tissue (Fig. 7). Analysis of the network interactions between DE miRNAs and their mRNA targets showed that the target with the highest score was FOXO3, which is a common target of all DE miRNAs (Fig. 3). FOXO transcription factors are part of the large Forkhead protein family, known for their role as transcriptional regulators characterized by a conserved DNA‐binding domain called the Forkhead box [28]. FOXO proteins came to prominence with the identification of chromosomal translocations in human tumors involving FOXO1, FOXO3, and FOXO4 [29]. These findings suggest a potentially important role for FOXO transcription factors in tumor development.

Altered expression of miR‐221‐3p and miR‐222‐3p has been associated with various tumors, suggesting their potential as biomarkers and therapeutic targets [30]. It appears that they may play an important regulatory role, either promoting or suppressing cancer [31]. Specifically, they can facilitate the transition from cell quiescence, promoting increased proliferation, survival, and metastatic potential, acting as oncogenes [32]. With regard to TGCTs, increased levels of these miRNAs have been detected in paraffin‐embedded formalin‐fixed tissues from seminomas compared to normal tissues; however, there are no evidence of their expression in these patients at seminal plasma level [33]. Our finding regarding the downregulation of these miRNAs in seminal plasma from TGCT patients are consistent with different studies showing an opposite expression trend between fluids and tissue [34]. To explain this, it has been hypothesized that in physiological conditions, cells tend to express miRNAs involved in normal homeostasis. If these cells become cancerous, they will tend to maintain inside miRNAs involved in cell proliferation, inhibiting cell death [35]. On the contrary, miRNAs highly expressed at the cellular level, may be secreted, since their function is disadvantageous for specific cell types in physiological or pathological conditions [34].

This hypothesis is supported by our data on differential analysis data, as we found that GRB10, SOCS3, and TICAM1 were down‐expressed in tumor tissue. They are all targets of miR‐221‐3p and miR‐222‐3p, which could confirm their upregulation in tumor tissue, which correspond to a downregulation of the circulating form. Unfortunately, we could not experimentally confirm their expression in spermatozoa, as these were collected at the IVF centre for the sole purpose of cryopreservation prior to chemotherapy. Our study showed a positive correlation between miR‐221‐3p and miR‐222‐3p expression levels in seminal plasma and sperm count. Previous studies have reported a downregulation of miR‐221‐3p in spermatozoa from oligospermic subjects compared to healthy men spermatozoa and a correlation between its expression levels and apoptotic factors, suggesting that miR‐221‐3p may be involved in proapoptotic pathways in spermatozoa [36]. Furthermore, *in vitro* studies have suggested that both miR‐221‐3p and miR‐222‐3p may play a critical role in maintaining the stem cell capacity of the undifferentiated spermatogonial population, and the downregulation of these miRNAs may be involved in the age‐related loss of germ line and may be an underlying cause of male infertility [37]. Our analysis showed that miR‐221‐3p was significantly downregulated in both TGCT patients and CTRL IS compared to CTRL NS. Its marked downregulation in the context of infertility suggests a possible role in this condition. In support of this hypothesis, our ROC curve analysis showed that this miRNA has good efficiency in discriminating CTRL IS from CTRL NS.

Our results showed that miR‐126‐3p was overexpressed in TGCTs compared to CTRLs. This miRNA is mainly expressed in endothelial cells, including capillaries and larger blood vessels, where it modulates angiogenesis by regulating a variety of transcripts [38]. It has been studied and correlated with several human cancers [39], but to date, there is no evidence of its involvement in TGCTs. Several studies suggest that miR‐126 may be a potential tumor suppressor in colorectal cancer and that its altered regulation may serve as a diagnostic biomarker as well as to distinguish metastatic from non‐metastatic colorectal cancer [40]. Less consistency is found in other tumor types, e.g., different studies have shown both up‐ and downregulation in tissues of in gastric, hepatocellular, and prostate cancers, suggesting that its regulation may vary depending on the expression of its target within the cells and their role [41, 42, 43]. In addition, miR‐126‐3p levels appear to be elevated in the serum of ovarian cancer patients compared to healthy women [44]. Similarly, miR‐126‐3p has been found to be overexpressed in the plasma of acute myeloid leukemia patients [45].

In breast cancer, miR‐126‐3p has been shown to directly interact with PIK3R2 in breast cancer, reducing its expression and thereby reducing resistance to trastuzumab [46]. Furthermore, in hepatocellular carcinoma cells, overexpression of miR‐126‐3p can significantly inhibit the PIK3R2/P‐AKT pathway, which is strongly associated with angiogenesis, revealing the anti‐angiogenic effects of miR‐126‐3p [47].

Among the common pathways of the identified miRNAs is the prolactin signaling pathway, in which both PIK3R2 and SOCS3 are involved. Prolactin can stimulate testicular functions, influence Leydig functions, increase the number of luteinizing hormone receptors, and promote steroidogenesis and androgen production [48]. In addition, a negative correlation between serum prolactin levels and sperm concentration has been demonstrated, suggesting that altered levels of this hormone may be associated with alterations in spermatogenesis [49]. Furthermore, altered expression levels of prolactin were associated with non‐seminomas [50]. Based on our results and the scientific literature collected and reported so far, we believe that the identified miRNAs could represent valuable noninvasive biomarkers in our model. This study certainly has some limitations, for instance, it would be useful to evaluate the differences in expression of the identified miRNAs and their targets also in spermatozoa from cancer patients and healthy subjects. Unfortunately, it is not possible for us to obtain spermatozoa from TGCT patients, since the plasma samples used in this study come from the IVF Centre of the Cannizzaro Hospital Cryopreservation center, where patients can preserve their gametes before starting cancer treatment. For this reason, each sample collected is precious and will only be used to preserve the fertility of the patients.

## 5 Conclusion

Our research has identified three miRNAs as significant biomarkers for TGCT. These miRNAs are easily detectable in seminal plasma, offering a promising noninvasive approach for the early diagnosis of TGCT. Importantly, infertility, which is frequently disregarded, is both a risk factor and an early indicator of testicular cancer. This connection underscores the potential of our findings in improving early detection strategies.

## Conflict of interest

SP is a shareholder and serves as the Operations Director of DESTINA Genomica SL.

## Author contributions

CDP, RB, SP, and CF contributed to conceptualization; CF, RB, AC, and NM contributed to methodology; CF, AC, AF, MS, and CB contributed to validation; CF, AC, and AF contributed to formal analysis and investigation; FL, MEV, PB, and PS contributed to resources; CF, CDP, and RB contributed to writing—original draft preparation; CF, RB, AC, DB, MR, SP, and CDP contributed to writing—review and editing; CF, RB, AC, AF, MS, CB, NM, FL, MEV, PB, PS, DB, MR, SP, and CDP contributed to visualization; CDP and RB contributed to supervision; CDP, MR, and SP contributed to funding acquisition. All authors have read and agreed to the published version of the manuscript.

## Supporting information


**Table S1.** miRCURY LNA miRNA serum/plasma focus PCR panel layout.

**Table S2.** Values of fold changes and *P*‐values for all miRNAs in Qiagen miRCURY LNA miRNA serum/plasma focus PCR panel.

**Table S3.** Values of fold changes and *P*‐values for differential expression analysis of GSE1818 dataset.

## Data Availability

The data that support the findings of this study are available from the corresponding author rosalia.battaglia@unict.it upon reasonable request.

## References

[1] Ferguson L , Agoulnik AI . Testicular cancer and cryptorchidism. Front Endocrinol (Lausanne). 2013;4:32. 10.3389/fendo.2013.00032 23519268 PMC3602796

[2] Brauner EV , Lim YH , Koch T , Uldbjerg CS , Gregersen LS , Pedersen MK , et al. Endocrine disrupting chemicals and risk of testicular cancer: a systematic review and meta‐analysis. J Clin Endocrinol Metab. 2021;106:e4834–e4860. 10.1210/clinem/dgab523 34270734 PMC8864757

[3] Cheng L , Lyu B , Roth LM . Perspectives on testicular germ cell neoplasms. Hum Pathol. 2017;59:10–25. 10.1016/j.humpath.2016.08.002 27569298

[4] Cheng L , Albers P , Berney DM , Feldman DR , Daugaard G , Gilligan T , et al. Testicular cancer. Nat Rev Dis Primers. 2018;4:29. 10.1038/s41572-018-0029-0 30291251

[5] Maiolino G , Fernandez‐Pascual E , Ochoa Arvizo MA , Vishwakarma R , Martinez‐Salamanca JI . Male infertility and the risk of developing testicular cancer: a critical contemporary literature review. Medicina (Kaunas). 2023;59:1305. 10.3390/medicina59071305 37512119 PMC10383207

[6] Hanson BM , Eisenberg ML , Hotaling JM . Male infertility: a biomarker of individual and familial cancer risk. Fertil Steril. 2018;109:6–19. 10.1016/j.fertnstert.2017.11.005 29307404

[7] Hotaling JM , Walsh TJ . Male infertility: a risk factor for testicular cancer. Nat Rev Urol. 2009;6:550–556. 10.1038/nrurol.2009.179 19724246

[8] Palermo G , Joris H , Devroey P , Van Steirteghem AC . Pregnancies after intracytoplasmic injection of single spermatozoon into an oocyte. Lancet. 1992;340:17–18. 10.1016/0140-6736(92)92425-f 1351601

[9] Gilligan TD , Seidenfeld J , Basch EM , Einhorn LH , Fancher T , Smith DC , et al. American Society of Clinical Oncology clinical practice guideline on uses of serum tumor markers in adult males with germ cell tumors. J Clin Oncol. 2010;28:3388–3404. 10.1200/JCO.2009.26.4481 20530278

[10] Lembeck AL , Puchas P , Hutterer G , Barth DA , Terbuch A , Bauernhofer T , et al. MicroRNAs as appropriate discriminators in non‐specific alpha‐fetoprotein (AFP) elevation in testicular germ cell tumor patients. Noncoding RNA. 2020;6:2. 10.3390/ncrna6010002 31906360 PMC7151547

[11] Mayor‐de‐Castro J , Aragon‐Chamizo J , Cano‐Velasco J , Hernandez‐Fernandez C . Biomarkers in testicular cancer. Arch Esp Urol. 2022;75:113–117. 35332880

[12] Menon A , Abd‐Aziz N , Khalid K , Poh CL , Naidu R . miRNA: a promising therapeutic target in cancer. Int J Mol Sci. 2022;23:11502. 10.3390/ijms231911502 36232799 PMC9569513

[13] Barchi M , Bielli P , Dolci S , Rossi P , Grimaldi P . Non‐coding RNAs and splicing activity in testicular germ cell tumors. Life (Basel). 2021;11:736. 10.3390/life11080736 34440480 PMC8399856

[14] Otmani K , Lewalle P . Tumor suppressor miRNA in cancer cells and the tumor microenvironment: mechanism of deregulation and clinical implications. Front Oncol. 2021;11:708765. 10.3389/fonc.2021.708765 34722255 PMC8554338

[15] Bahmyari S , Khatami SH , Taghvimi S , Rezaei Arablouydareh S , Taheri‐Anganeh M , Ghasemnejad‐Berenji H , et al. MicroRNAs in male fertility. DNA Cell Biol. 2024;43:108–124. 10.1089/dna.2023.0314 38394131

[16] Bjorkgren I , Gylling H , Turunen H , Huhtaniemi I , Strauss L , Poutanen M , et al. Imbalanced lipid homeostasis in the conditional Dicer1 knockout mouse epididymis causes instability of the sperm membrane. FASEB J. 2015;29:433–442. 10.1096/fj.14-259382 25366345

[17] Papaioannou MD , Lagarrigue M , Vejnar CE , Rolland AD , Kuhne F , Aubry F , et al. Loss of dicer in sertoli cells has a major impact on the testicular proteome of mice. Mol Cell Proteomics. 2011;10:M900587MCP200. 10.1074/mcp.M900587-MCP200 20467044 PMC3069350

[18] Korhonen HM , Meikar O , Yadav RP , Papaioannou MD , Romero Y , Da Ros M , et al. Dicer is required for haploid male germ cell differentiation in mice. PLoS One. 2011;6:e24821. 10.1371/journal.pone.0024821 21949761 PMC3174967

[19] Wu W , Qin Y , Li Z , Dong J , Dai J , Lu C , et al. Genome‐wide microRNA expression profiling in idiopathic non‐obstructive azoospermia: significant up‐regulation of miR‐141, miR‐429 and miR‐7‐1‐3p. Hum Reprod. 2013;28:1827–1836. 10.1093/humrep/det099 23559187

[20] Wang C , Yang C , Chen X , Yao B , Yang C , Zhu C , et al. Altered profile of seminal plasma microRNAs in the molecular diagnosis of male infertility. Clin Chem. 2011;57:1722–1731. 10.1373/clinchem.2011.169714 21933900

[21] Pelloni M , Coltrinari G , Paoli D , Pallotti F , Lombardo F , Lenzi A , et al. Differential expression of miRNAs in the seminal plasma and serum of testicular cancer patients. Endocrine. 2017;57:518–527. 10.1007/s12020-016-1150-z 27796811

[22] Khawar MB , Mehmood R , Roohi N . MicroRNAs: recent insights towards their role in male infertility and reproductive cancers. Bosn J Basic Med Sci. 2019;19:31–42. 10.17305/bjbms.2018.3477 30599090 PMC6387678

[23] Huang HY , Lin YC , Cui S , Huang Y , Tang Y , Xu J , et al. miRTarBase update 2022: an informative resource for experimentally validated miRNA‐target interactions. Nucleic Acids Res. 2022;50:D222–D230. 10.1093/nar/gkab1079 34850920 PMC8728135

[24] Licursi V , Conte F , Fiscon G , Paci P . MIENTURNET: an interactive web tool for microRNA‐target enrichment and network‐based analysis. BMC Bioinformatics. 2019;20:545. 10.1186/s12859-019-3105-x 31684860 PMC6829817

[25] Liu C , Ma Y , Zhao J , Nussinov R , Zhang Y‐C , Cheng F , et al. Computational network biology: data, models, and applications. Phys Rep. 2020;846:1–66. 10.1016/j.physrep.2019.12.004

[26] Chakrabortty A , Patton DJ , Smith BF , Agarwal P . miRNAs: potential as biomarkers and therapeutic targets for cancer. Genes (Basel). 2023;14:1375. 10.3390/genes14071375 37510280 PMC10378777

[27] Nestler T , Schoch J , Belge G , Dieckmann KP . MicroRNA‐371a‐3p‐the novel serum biomarker in testicular germ cell tumors. Cancers (Basel). 2023;15:3944. 10.3390/cancers15153944 37568759 PMC10417034

[28] Link W . Introduction to FOXO biology. Methods Mol Biol. 2019;1890:1–9. 10.1007/978-1-4939-8900-3_1 30414140

[29] Wang Y , Zhou Y , Graves DT . FOXO transcription factors: their clinical significance and regulation. Biomed Res Int. 2014;2014:925350. 10.1155/2014/925350 24864265 PMC4016844

[30] Garofalo M , Quintavalle C , Romano G , Croce CM , Condorelli G . miR221/222 in cancer: their role in tumor progression and response to therapy. Curr Mol Med. 2012;12:27–33. 10.2174/156652412798376170 22082479 PMC3673714

[31] Song Q , An Q , Niu B , Lu X , Zhang N , Cao X . Role of miR‐221/222 in tumor development and the underlying mechanism. J Oncol. 2019;2019:7252013. 10.1155/2019/7252013 31929798 PMC6942871

[32] Di Martino MT , Arbitrio M , Caracciolo D , Cordua A , Cuomo O , Grillone K , et al. miR‐221/222 as biomarkers and targets for therapeutic intervention on cancer and other diseases: a systematic review. Mol Ther Nucleic Acids. 2022;27:1191–1224. 10.1016/j.omtn.2022.02.005 35282417 PMC8891816

[33] Bing Z , Master SR , Tobias JW , Baldwin DA , Xu XW , Tomaszewski JE . MicroRNA expression profiles of seminoma from paraffin‐embedded formalin‐fixed tissue. Virchows Arch. 2012;461:663–668. 10.1007/s00428-012-1325-9 23064661

[34] Ragusa M , Barbagallo C , Cirnigliaro M , Battaglia R , Brex D , Caponnetto A , et al. Asymmetric RNA distribution among cells and their secreted exosomes: biomedical meaning and considerations on diagnostic applications. Front Mol Biosci. 2017;4:66. 10.3389/fmolb.2017.00066 29046875 PMC5632685

[35] Kanlikilicer P , Rashed MH , Bayraktar R , Mitra R , Ivan C , Aslan B , et al. Ubiquitous release of exosomal tumor suppressor miR‐6126 from ovarian cancer cells. Cancer Res. 2016;76:7194–7207. 10.1158/0008-5472.CAN-16-0714 27742688 PMC5901763

[36] Saadh MJ , Pecho RDC , Jamal A , Alothaim AS , Kamal MA , Warsi MK , et al. Reduced expression of miR‐221 is associated with the pro‐apoptotic pathways in spermatozoa of oligospermia men. J Reprod Immunol. 2023;160:104159. 10.1016/j.jri.2023.104159 37913711

[37] Yang QE , Racicot KE , Kaucher AV , Oatley MJ , Oatley JM . MicroRNAs 221 and 222 regulate the undifferentiated state in mammalian male germ cells. Development. 2013;140:280–290. 10.1242/dev.087403 23221369 PMC3597206

[38] Fish JE , Santoro MM , Morton SU , Yu S , Yeh RF , Wythe JD , et al. miR‐126 regulates angiogenic signaling and vascular integrity. Dev Cell. 2008;15:272–284. 10.1016/j.devcel.2008.07.008 18694566 PMC2604134

[39] Merulla AE , Stella M , Barbagallo C , Battaglia R , Caponnetto A , Broggi G , et al. circSMARCA5 is an upstream regulator of the expression of miR‐126‐3p, miR‐515‐5p, and their mRNA targets, insulin‐like growth factor binding protein 2 (IGFBP2) and NRAS proto‐oncogene, GTPase (NRAS) in glioblastoma. Int J Mol Sci. 2022;23:13676. 10.3390/ijms232213676 36430152 PMC9690846

[40] Huang W , Lin J , Zhang H . miR‐126: a novel regulator in colon cancer. Biomed Rep. 2016;4:131–134. 10.3892/br.2015.549 26893826 PMC4734020

[41] Otsubo T , Akiyama Y , Hashimoto Y , Shimada S , Goto K , Yuasa Y . MicroRNA‐126 inhibits SOX2 expression and contributes to gastric carcinogenesis. PLoS One. 2011;6:e16617. 10.1371/journal.pone.0016617 21304604 PMC3029394

[42] Barshack I , Meiri E , Rosenwald S , Lebanony D , Bronfeld M , Aviel‐Ronen S , et al. Differential diagnosis of hepatocellular carcinoma from metastatic tumors in the liver using microRNA expression. Int J Biochem Cell Biol. 2010;42:1355–1362. 10.1016/j.biocel.2009.02.021 20619223

[43] Watahiki A , Wang Y , Morris J , Dennis K , O'Dwyer HM , Gleave M , et al. MicroRNAs associated with metastatic prostate cancer. PLoS One. 2011;6:e24950. 10.1371/journal.pone.0024950 21980368 PMC3184096

[44] Resnick KE , Alder H , Hagan JP , Richardson DL , Croce CM , Cohn DE . The detection of differentially expressed microRNAs from the serum of ovarian cancer patients using a novel real‐time PCR platform. Gynecol Oncol. 2009;112:55–59. 10.1016/j.ygyno.2008.08.036 18954897

[45] Almohsen F , Al‐Rubaie HA , Habib MA , Nasr SA , Perni R , Al‐Quraishi L . Circulating miR‐126‐3p and miR‐423‐5p expression in de novo adult acute myeloid leukemia: correlations with response to induction therapy and the 2‐year overall survival. J Blood Med. 2022;13:83–92. 10.2147/JBM.S347397 35210895 PMC8863343

[46] Fu R , Tong JS . miR‐126 reduces trastuzumab resistance by targeting PIK3R2 and regulating AKT/mTOR pathway in breast cancer cells. J Cell Mol Med. 2020;24:7600–7608. 10.1111/jcmm.15396 32410348 PMC7339158

[47] Du C , Lv Z , Cao L , Ding C , Gyabaah OA , Xie H , et al. MiR‐126‐3p suppresses tumor metastasis and angiogenesis of hepatocellular carcinoma by targeting LRP6 and PIK3R2. J Transl Med. 2014;12:259. 10.1186/s12967-014-0259-1 25240815 PMC4189615

[48] Rastrelli G , Corona G , Maggi M . The role of prolactin in andrology: what is new? Rev Endocr Metab Disord. 2015;16:233–248. 10.1007/s11154-015-9322-3 26542707

[49] Spaggiari G , Costantino F , Granata ARM , Tagliavini S , Canu G , Varani M , et al. Prolactin and spermatogenesis: new lights on the interplay between prolactin and sperm parameters. Endocrine. 2023;81:330–339. 10.1007/s12020-023-03375-x 37140814

[50] Torzsok P , Oswald D , Dieckmann KP , Angerer M , Scherer LC , Tymoszuk P , et al. Subsets of preoperative sex hormones in testicular germ cell cancer: a retrospective multicenter study. Sci Rep. 2023;13:14604. 10.1038/s41598-023-41915-7 37669975 PMC10480169

