# Supplementary material for: MicroRNAs in seminal plasma are able to discern infertile men at increased risk of developing testicular cancer
Source: Mol Oncol. 2024 Dec 16;19(4):1188–202. doi: 10.1002/1878-0261.13784 (PMC11977642; doi:10.1002/1878-0261.13784)
Supplement: Supplementary file 1 — Table S1. miRCURY LNA miRNA serum/plasma focus PCR panel layout. [file MOL2-19-1188-s002.docx]

**Supplementary Table 1. miRCURY LNA miRNA Serum/Plasma Focus PCR Panel layout.**

| **miRname (human)** | **Panel plate position** | **microRNA target sequence** | **Assay type** |
| --- | --- | --- | --- |
| hsa-let-7a-5p | A01 | UGAGGUAGUAGGUUGUAUAGUU | GOI |
| hsa-miR-1-3p | A02 | UGGAAUGUAAAGAAGUAUGUAU | GOI |
| hsa-miR-100-5p | A03 | AACCCGUAGAUCCGAACUUGUG | GOI |
| hsa-miR-106b-5p | A04 | UAAAGUGCUGACAGUGCAGAU | GOI |
| hsa-miR-10b-5p | A05 | UACCCUGUAGAACCGAAUUUGUG | GOI |
| hsa-miR-122-5p | A06 | UGGAGUGUGACAAUGGUGUUUG | GOI |
| hsa-miR-124-3p | A07 | UAAGGCACGCGGUGAAUGCC | GOI |
| hsa-miR-125b-5p | A08 | UCCCUGAGACCCUAACUUGUGA | GOI |
| hsa-miR-126-3p | A09 | UCGUACCGUGAGUAAUAAUGCG | GOI |
| hsa-miR-133a-3p | A10 | UUUGGUCCCCUUCAACCAGCUG | GOI |
| hsa-miR-133b | A11 | UUUGGUCCCCUUCAACCAGCUA | GOI |
| hsa-miR-134-5p | A12 | UGUGACUGGUUGACCAGAGGGG | GOI |
| hsa-miR-141-3p | B01 | UAACACUGUCUGGUAAAGAUGG | GOI |
| hsa-miR-143-3p | B02 | UGAGAUGAAGCACUGUAGCUC | GOI |
| hsa-miR-146a-5p | B03 | UGAGAACUGAAUUCCAUGGGUU | GOI |
| hsa-miR-150-5p | B04 | UCUCCCAACCCUUGUACCAGUG | GOI |
| hsa-miR-155-5p | B05 | UUAAUGCUAAUCGUGAUAGGGGU | GOI |
| hsa-miR-17-5p | B06 | CAAAGUGCUUACAGUGCAGGUAG | GOI |
| hsa-miR-17-3p | B07 | ACUGCAGUGAAGGCACUUGUAG | GOI |
| hsa-miR-18a-5p | B08 | UAAGGUGCAUCUAGUGCAGAUAG | GOI |
| hsa-miR-192-5p | B09 | CUGACCUAUGAAUUGACAGCC | GOI |
| hsa-miR-195-5p | B10 | UAGCAGCACAGAAAUAUUGGC | GOI |
| hsa-miR-196a-5p | B11 | UAGGUAGUUUCAUGUUGUUGGG | GOI |
| hsa-miR-19a-3p | B12 | UGUGCAAAUCUAUGCAAAACUGA | GOI |
| hsa-miR-19b-3p | C01 | UGUGCAAAUCCAUGCAAAACUGA | GOI |
| hsa-miR-200a-3p | C02 | UAACACUGUCUGGUAACGAUGU | GOI |
| hsa-miR-200b-3p | C03 | UAAUACUGCCUGGUAAUGAUGA | GOI |
| hsa-miR-200c-3p | C04 | UAAUACUGCCGGGUAAUGAUGGA | GOI |
| hsa-miR-203a-3p | C05 | GUGAAAUGUUUAGGACCACUAG | GOI |
| hsa-miR-205-5p | C06 | UCCUUCAUUCCACCGGAGUCUG | GOI |
| hsa-miR-208a-3p | C07 | AUAAGACGAGCAAAAAGCUUGU | GOI |
| hsa-miR-20a-5p | C08 | UAAAGUGCUUAUAGUGCAGGUAG | GOI |
| hsa-miR-21-5p | C09 | UAGCUUAUCAGACUGAUGUUGA | GOI |
| hsa-miR-210-3p | C10 | CUGUGCGUGUGACAGCGGCUGA | GOI |
| hsa-miR-214-3p | C11 | ACAGCAGGCACAGACAGGCAGU | GOI |
| hsa-miR-215-5p | C12 | AUGACCUAUGAAUUGACAGAC | GOI |
| hsa-miR-221-3p | D01 | AGCUACAUUGUCUGCUGGGUUUC | GOI |
| hsa-miR-222-3p | D02 | AGCUACAUCUGGCUACUGGGU | GOI |
| hsa-miR-223-3p | D03 | UGUCAGUUUGUCAAAUACCCCA | GOI |
| hsa-miR-224-5p | D04 | CAAGUCACUAGUGGUUCCGUU | GOI |
| hsa-miR-23a-3p | D05 | AUCACAUUGCCAGGGAUUUCC | GOI |
| hsa-miR-25-3p | D06 | CAUUGCACUUGUCUCGGUCUGA | GOI |
| hsa-miR-27a-3p | D07 | UUCACAGUGGCUAAGUUCCGC | GOI |
| hsa-miR-296-5p | D08 | AGGGCCCCCCCUCAAUCCUGU | GOI |
| hsa-miR-29a-3p | D09 | UAGCACCAUCUGAAAUCGGUUA | GOI |
| hsa-miR-30d-5p | D10 | UGUAAACAUCCCCGACUGGAAG | GOI |
| hsa-miR-34a-5p | D11 | UGGCAGUGUCUUAGCUGGUUGU | GOI |
| hsa-miR-375 | D12 | UUUGUUCGUUCGGCUCGCGUGA | GOI |
| hsa-miR-423-5p | E01 | UGAGGGGCAGAGAGCGAGACUUU | GOI |
| hsa-miR-499a-5p | E02 | UUAAGACUUGCAGUGAUGUUU | GOI |
| hsa-miR-574-3p | E03 | CACGCUCAUGCACACACCCACA | GOI |
| hsa-miR-885-5p | E04 | UCCAUUACACUACCCUGCCUCU | GOI |
| hsa-miR-9-5p | E05 | UCUUUGGUUAUCUAGCUGUAUGA | GOI |
| hsa-miR-92a-3p | E06 | UAUUGCACUUGUCCCGGCCUGU | GOI |
| hsa-miR-93-5p | E07 | CAAAGUGCUGUUCGUGCAGGUAG | GOI |
| hsa-let-7c-5p | E08 | UGAGGUAGUAGGUUGUAUGGUU | GOI |
| hsa-miR-107 | E09 | AGCAGCAUUGUACAGGGCUAUCA | GOI |
| hsa-miR-10a-5p | E10 | UACCCUGUAGAUCCGAAUUUGUG | GOI |
| hsa-miR-128-3p | E11 | UCACAGUGAACCGGUCUCUUU | GOI |
| hsa-miR-130b-3p | E12 | CAGUGCAAUGAUGAAAGGGCAU | GOI |
| hsa-miR-145-5p | F01 | GUCCAGUUUUCCCAGGAAUCCCU | GOI |
| hsa-miR-148a-3p | F02 | UCAGUGCACUACAGAACUUUGU | GOI |
| hsa-miR-15a-5p | F03 | UAGCAGCACAUAAUGGUUUGUG | GOI |
| hsa-miR-184 | F04 | UGGACGGAGAACUGAUAAGGGU | GOI |
| hsa-miR-193a-5p | F05 | UGGGUCUUUGCGGGCGAGAUGA | GOI |
| hsa-miR-204-5p | F06 | UUCCCUUUGUCAUCCUAUGCCU | GOI |
| hsa-miR-206 | F07 | UGGAAUGUAAGGAAGUGUGUGG | GOI |
| hsa-miR-211-5p | F08 | UUCCCUUUGUCAUCCUUCGCCU | GOI |
| hsa-miR-26b-5p | F09 | UUCAAGUAAUUCAGGAUAGGU | GOI |
| hsa-miR-30e-5p | F10 | UGUAAACAUCCUUGACUGGAAG | GOI |
| hsa-miR-372-3p | F11 | AAAGUGCUGCGACAUUUGAGCGU | GOI |
| hsa-miR-373-3p | F12 | GAAGUGCUUCGAUUUUGGGGUGU | GOI |
| hsa-miR-374a-5p | G01 | UUAUAAUACAACCUGAUAAGUG | GOI |
| hsa-miR-376c-3p | G02 | AACAUAGAGGAAAUUCCACGU | GOI |
| hsa-miR-7-5p | G03 | UGGAAGACUAGUGAUUUUGUUGU | GOI |
| hsa-miR-96-5p | G04 | UUUGGCACUAGCACAUUUUUGCU | GOI |
| hsa-miR-103a-3p | G05 | AGCAGCAUUGUACAGGGCUAUGA | GOI |
| hsa-miR-15b-5p | G06 | UAGCAGCACAUCAUGGUUUACA | GOI |
| hsa-miR-16-5p | G07 | UAGCAGCACGUAAAUAUUGGCG | GOI |
| hsa-miR-191-5p | G08 | CAACGGAAUCCCAAAAGCAGCUG | GOI |
| hsa-miR-22-3p | G09 | AAGCUGCCAGUUGAAGAACUGU | GOI |
| hsa-miR-24-3p | G10 | UGGCUCAGUUCAGCAGGAACAG | GOI |
| hsa-miR-26a-5p | G11 | UUCAAGUAAUCCAGGAUAGGCU | GOI |
| hsa-miR-31-5p | G12 | AGGCAAGAUGCUGGCAUAGCU | GOI |
| cel-miR-39-3p | H01 |  | Spike |
| cel-miR-39-3p | H02 |  | Spike |
| hsa-miR-30c-5p | H03 |  | Ref |
| hsa-miR-103a-3p | H04 |  | Ref |
| hsa-miR-451a | H05 |  | Ref |
| hsa-miR-23a-3p | H06 |  | Ref |
| UniSp2 | H07 |  | Spike |
| UniSp4 | H08 |  | Spike |
| UniSp5 | H09 |  | Spike |
| UniSp6 | H10 |  | Spike |
| UniSp3 IPC | H11 |  | IPC |
| UniSp3 IPC | H12 |  | IPC |
