# Supplementary material for: MicroRNAs in seminal plasma are able to discern infertile men at increased risk of developing testicular cancer
Source: Mol Oncol. 2024 Dec 16;19(4):1188–202. doi: 10.1002/1878-0261.13784 (PMC11977642; doi:10.1002/1878-0261.13784)
Supplement: Supplementary file 2 — Table S2. Values of fold changes and P‐values for all miRNAs in Qiagen miRCURY LNA miRNA serum/plasma focus PCR panel. [file MOL2-19-1188-s001.docx]

**Supplementary Table 2. Values of Fold-changes and p-values for all miRNAs in Qiagen miRCURY LNA miRNA Serum/Plasma Focus PCR Panel.** TGCT= patients affected by Testicular Germ Cell Tumors; CTRL IS= healthy subjects with impaired spermiogram; CTRL NS= healthy subjects with normal spermiogram. Fold-changes are expressed as 2^-∆∆Ct^.

|  | **TGCT vs CTRL IS** | | |  | **TGCT vs CTRL NS** | | |  | **CTRL IS vs CTRL NS** | | | |
| --- | --- | --- | --- | --- | --- | --- | --- | --- | --- | --- | --- | --- |
|  |  | | |  |  | | |  |  | | | |
| **miRNAs** | **FC** | **p-values** |  | | | **FC** | **p-values** |  | | **FC** | **p-values** |  |
|  |  |  |  | | |  |  |  | |  |  |  |
| hsa-let-7a-5p | 2.343 | 0.072 |  | | | 0.932 | 0.850 |  | | 0.398 | 0.002 |  |
| hsa-let-7c-5p | 3.465 | 0.076 |  | | | 1.277 | 0.653 |  | | 0.369 | 0.002 |  |
| hsa-miR-100-5p | 0.690 | 0.411 |  | | | 0.468 | 0.183 |  | | 0.679 | 0.184 |  |
| hsa-miR-103a-3p | 2.894 | 0.135 |  | | | 0.737 | 0.267 |  | | 0.255 | 0.071 |  |
| hsa-miR-106b-5p | 2.021 | 0.302 |  | | | 0.869 | 0.558 |  | | 0.430 | 0.247 |  |
| hsa-miR-107 | 1.712 | 0.218 |  | | | 1.261 | 0.519 |  | | 0.737 | 0.184 |  |
| hsa-miR-10a-5p | 0.496 | 0.326 |  | | | 0.318 | 0.148 |  | | 0.642 | 0.033 |  |
| hsa-miR-10b-5p | 0.758 | 0.473 |  | | | 0.240 | 0.008 |  | | 0.316 | 0.008 |  |
| hsa-miR-122-5p | 0.033 | 0.017 |  | | | 0.031 | 0.017 |  | | 0.943 | 0.786 |  |
| hsa-miR-124-3p | 1.402 | 0.773 |  | | | 1.282 | 0.766 |  | | 0.915 | 0.928 |  |
| hsa-miR-125-5p | 1.230 | 0.556 |  | | | 0.539 | 0.121 |  | | 0.438 | 0.007 |  |
| **hsa-miR-126-3p** | 5.081 | 0.048 |  | | | 1.242 | 0.169 |  | | 0.244 | 0.071 |  |
| hsa-miR-128-3p | 1.648 | 0.486 |  | | | 0.740 | 0.439 |  | | 0.449 | 0.226 |  |
| hsa-miR-130b-3p | 1.076 | 0.612 |  | | | 0.904 | 0.509 |  | | 0.840 | 0.368 |  |
| hsa-miR-133a-3p | 0.101 | 0.072 |  | | | 0.237 | 0.032 |  | | 2.356 | 0.379 |  |
| hsa-miR-133b | 0.101 | 0.072 |  | | | 0.237 | 0.032 |  | | 2.356 | 0.379 |  |
| hsa-miR-134-5p | 1.537 | 0.742 |  | | | 3.620 | 0.221 |  | | 2.356 | 0.379 |  |
| hsa-miR-1-3p | 0.101 | 0.119 |  | | | 0.950 | 0.952 |  | | 9.425 | 0.061 |  |
| hsa-miR-141-3p | 0.633 | 0.375 |  | | | 0.620 | 0.251 |  | | 0.978 | 0.961 |  |
| hsa-miR-143-3p | 0.370 | 0.356 |  | | | 1.741 | 0.487 |  | | 4.713 | 0.206 |  |
| hsa-miR-145-5p | 0.225 | 0.270 |  | | | 0.280 | 0.259 |  | | 1.246 | 0.838 |  |
| hsa-miR-146a-5p | 2.517 | 0.441 |  | | | 0.870 | 0.901 |  | | 0.346 | 0.050 |  |
| hsa-miR-148a-3p | 5.050 | 0.146 |  | | | 1.139 | 0.865 |  | | 0.226 | 0.057 |  |
| hsa-miR-150-5p | 0.442 | 0.244 |  | | | 0.395 | 0.196 |  | | 0.895 | 0.428 |  |
| hsa-miR-155-5p | 2.673 | 0.314 |  | | | 0.625 | 0.018 |  | | 0.234 | 0.163 |  |
| hsa-miR-15a-5p | 1.460 | 0.473 |  | | | 0.592 | 0.203 |  | | 0.406 | 0.147 |  |
| hsa-miR-15b-5p | 1.969 | 0.490 |  | | | 1.765 | 0.545 |  | | 0.896 | 0.788 |  |
| hsa-miR-16-5p | 2.096 | 0.167 |  | | | 0.671 | 0.002 |  | | 0.320 | 0.060 |  |
| hsa-miR-17-3p | 0.146 | 0.072 |  | | | 0.688 | 0.510 |  | | 4.721 | 0.070 |  |
| hsa-miR-17-5p | 2.441 | 0.155 |  | | | 1.225 | 0.418 |  | | 0.502 | 0.223 |  |
| hsa-miR-184 | 0.032 | 0.014 |  | | | 0.139 | 0.108 |  | | 4.386 | 0.139 |  |
| hsa-miR-18a-5p | 1.467 | 0.303 |  | | | 0.841 | 0.584 |  | | 0.573 | 0.141 |  |
| hsa-miR-191-5p | 1.224 | 0.579 |  | | | 0.959 | 0.909 |  | | 0.784 | 0.011 |  |
| hsa-miR-192-5p | 2.050 | 0.199 |  | | | 0.732 | 0.424 |  | | 0.357 | 0.034 |  |
| hsa-miR-193a-5p | 0.595 | 0.348 |  | | | 1.044 | 0.918 |  | | 1.754 | 0.153 |  |
| hsa-miR-195-5p | 0.954 | 0.945 |  | | | 1.322 | 0.688 |  | | 1.386 | 0.292 |  |
| hsa-miR-196a-5p | 0.393 | 0.585 |  | | | 1.850 | 0.692 |  | | 4.713 | 0.206 |  |
| hsa-miR-19a-3p | 13.534 | 0.205 |  | | | 1.339 | 0.636 |  | | 0.099 | 0.232 |  |
| hsa-miR-19b-3p | 3.438 | 0.326 |  | | | 0.676 | 0.072 |  | | 0.197 | 0.215 |  |
| hsa-miR-200a-3p | 1.579 | 0.484 |  | | | 0.616 | 0.090 |  | | 0.391 | 0.209 |  |
| hsa-miR-200b-3p | 2.807 | 0.145 |  | | | 0.779 | 0.102 |  | | 0.277 | 0.091 |  |
| hsa-miR-200c-3p | 1.276 | 0.423 |  | | | 0.708 | 0.319 |  | | 0.555 | 0.021 |  |
| hsa-miR-203a-3p | 2.071 | 0.050 |  | | | 0.696 | 0.179 |  | | 0.336 | 0.004 |  |
| hsa-miR-204-5p | 0.593 | 0.123 |  | | | 0.242 | 0.007 |  | | 0.409 | 0.017 |  |
| hsa-miR-205-5p | 0.325 | 0.245 |  | | | 0.263 | 0.179 |  | | 0.809 | 0.260 |  |
| hsa-miR-206 | 0.063 | 0.065 |  | | | 0.598 | 0.509 |  | | 9.425 | 0.061 |  |
| hsa-miR-208a-3p | 0.157 | 0.462 |  | | | 2.952 | 0.655 |  | | 18.850 | 0.004 |  |
| hsa-miR-20a-5p | 1.913 | 0.419 |  | | | 0.954 | 0.901 |  | | 0.499 | 0.342 |  |
| hsa-miR-210-3p | 0.462 | 0.044 |  | | | 0.622 | 0.071 |  | | 1.346 | 0.220 |  |
| hsa-miR-211-5p | 0.141 | 0.308 |  | | | 1.326 | 0.856 |  | | 9.425 | 0.061 |  |
| hsa-miR-214-3p | 1.313 | 0.803 |  | | | 3.261 | 0.114 |  | | 2.485 | 0.351 |  |
| hsa-miR-215-5p | 0.770 | 0.363 |  | | | 0.718 | 0.174 |  | | 0.933 | 0.687 |  |
| hsa-miR-21-5p | 2.801 | 0.186 |  | | | 1.004 | 0.982 |  | | 0.358 | 0.183 |  |
| **hsa-miR-221-3p** | 0.332 | 0.057 |  | | | 0.132 | 0.008 |  | | 0.397 | 0.001 |  |
| **hsa-miR-222-3p** | 0.167 | 0.001 |  | | | 0.115 | 0.001 |  | | 0.687 | 0.075 |  |
| hsa-miR-223-3p | 1.889 | 0.677 |  | | | 1.480 | 0.796 |  | | 0.783 | 0.022 |  |
| hsa-miR-22-3p | 0.700 | 0.336 |  | | | 1.139 | 0.750 |  | | 1.627 | 0.138 |  |
| hsa-miR-224-5p | 0.384 | 0.366 |  | | | 0.309 | 0.276 |  | | 0.805 | 0.286 |  |
| hsa-miR-23a-3p | 2.272 | 0.271 |  | | | 0.557 | 0.028 |  | | 0.245 | 0.086 |  |
| hsa-miR-24-3p | 2.315 | 0.054 |  | | | 0.873 | 0.474 |  | | 0.377 | 0.051 |  |
| hsa-miR-25-3p | 0.860 | 0.638 |  | | | 0.737 | 0.352 |  | | 0.857 | 0.185 |  |
| hsa-miR-26a-5p | 8.897 | 0.158 |  | | | 1.646 | 0.622 |  | | 0.185 | 0.127 |  |
| hsa-miR-26b-5p | 21.658 | 0.080 |  | | | 2.948 | 0.326 |  | | 0.136 | 0.092 |  |
| hsa-miR-27a-3p | 3.692 | 0.127 |  | | | 1.249 | 0.593 |  | | 0.338 | 0.127 |  |
| hsa-miR-296-5p | 1.059 | 0.936 |  | | | 0.255 | 0.118 |  | | 0.241 | 0.001 |  |
| hsa-miR-30c-5p | 0.880 | 0.836 |  | | | 0.476 | 0.280 |  | | 0.541 | 0.011 |  |
| hsa-miR-30d-5p | 1.074 | 0.678 |  | | | 0.765 | 0.212 |  | | 0.712 | 0.036 |  |
| hsa-miR-30e-5p | 5.295 | 0.193 |  | | | 1.200 | 0.659 |  | | 0.227 | 0.220 |  |
| hsa-miR-31-5p | 1.108 | 0.930 |  | | | 1.018 | 0.987 |  | | 0.919 | 0.773 |  |
| hsa-miR-34a-5p | 1.308 | 0.442 |  | | | 1.097 | 0.732 |  | | 0.838 | 0.545 |  |
| hsa-miR-372-3p | 0.063 | 0.065 |  | | | 0.033 | 0.011 |  | | 0.526 | 0.523 |  |
| hsa-miR-373-3p | 0.567 | 0.600 |  | | | 0.331 | 0.112 |  | | 0.584 | 0.567 |  |
| hsa-miR-374a-5p | 10.298 | 0.179 |  | | | 1.140 | 0.885 |  | | 0.111 | 0.144 |  |
| hsa-miR-375 | 1.075 | 0.787 |  | | | 0.766 | 0.361 |  | | 0.712 | 0.006 |  |
| hsa-miR-376c-3p | 1.245 | 0.732 |  | | | 0.990 | 0.986 |  | | 0.796 | 0.483 |  |
| hsa-miR-423-5p | 0.586 | 0.203 |  | | | 0.871 | 0.581 |  | | 1.486 | 0.266 |  |
| hsa-miR-499a-5p | 0.286 | 0.219 |  | | | 1.450 | 0.167 |  | | 5.074 | 0.135 |  |
| hsa-miR-574-3p | 0.612 | 0.242 |  | | | 0.588 | 0.193 |  | | 0.961 | 0.745 |  |
| hsa-miR-7-5p | 3.078 | 0.113 |  | | | 1.840 | 0.290 |  | | 0.598 | 0.237 |  |
| hsa-miR-885-5p | 0.080 | 0.060 |  | | | 0.086 | 0.089 |  | | 1.073 | 0.935 |  |
| hsa-miR-92a-3p | 1.479 | 0.344 |  | | | 0.592 | 0.000 |  | | 0.400 | 0.065 |  |
| hsa-miR-93-5p | 1.205 | 0.505 |  | | | 0.888 | 0.662 |  | | 0.737 | 0.003 |  |
| hsa-miR-9-5p | 0.142 | 0.109 |  | | | 0.123 | 0.071 |  | | 0.869 | 0.788 |  |
| hsa-miR-96-5p | 6.605 | 0.221 |  | | | 0.623 | 0.125 |  | | 0.094 | 0.143 |  |
