# Supplementary material for: MicroRNAs in seminal plasma are able to discern infertile men at increased risk of developing testicular cancer
Source: Mol Oncol. 2024 Dec 16;19(4):1188–202. doi: 10.1002/1878-0261.13784 (PMC11977642; doi:10.1002/1878-0261.13784)
Supplement: Supplementary file 3 — Table S3. Values of fold changes and P‐values for differential expression analysis of GSE1818 dataset. [file MOL2-19-1188-s003.docx]

**Supplementary Table 3.** **Values of Fold-changes and p-values for differential expression analysis of GSE1818 dataset.** Fold-changes are expressed as log_2_FC.

| **Genes** | **FC** | **p-values adj** |
| --- | --- | --- |
|  |  |  |
| PIK3R2 | -2.546 | 4.64E-06 |
| SOCS3 | -2.213 | 9.43E-05 |
| GRB10 | -2.115 | 0.00238234 |
| CXCR4 | 2.387 | 0.01150379 |
| TICAM1 | -1.202 | 0.01694361 |
| IGFBP2 | -2.581 | 0.02675266 |
| RANBP2 | -6.784 | 1.05E-13 |
| ALG6 | -6.887 | 4.97E-12 |
| ISG20L2 | -3.635 | 4.97E-12 |
| C8orf14 | -6.295 | 7.64E-12 |
| PEX12 | -5.660 | 7.64E-12 |
| BCS1L | -8.089 | 1.58E-11 |
| TNPO3 | -6.692 | 2.55E-11 |
| MS4A5 | -3.996 | 2.55E-11 |
| ACADS | -5.514 | 2.69E-11 |
| LOC92345 | -5.036 | 1.35E-10 |
| RDHE2 | -4.361 | 1.64E-10 |
| ZNF461 | -5.165 | 4.56E-10 |
| GPR174 | -4.773 | 4.87E-10 |
| GCET2 | -3.349 | 5.27E-10 |
| SCYL1 | -6.525 | 6.62E-10 |
| MTIF3 | -5.380 | 7.30E-10 |
| TYR | -5.514 | 7.53E-10 |
| CITED1 | -5.466 | 1.05E-09 |
| SELE | -4.346 | 1.17E-09 |
| ADORA1 | -3.917 | 1.17E-09 |
| PPID | -3.598 | 1.31E-09 |
| CASP14 | -5.555 | 1.34E-09 |
| PPT2 | -3.665 | 1.34E-09 |
| TSHZ2 | -4.417 | 1.34E-09 |
| MGC2749 | -3.674 | 1.56E-09 |
| CLDN23 | -3.899 | 1.61E-09 |
| C22orf31 | -2.853 | 1.68E-09 |
| RAB14 | -6.472 | 2.02E-09 |
| KIAA2010 | -5.429 | 2.02E-09 |
| APH1B | -6.211 | 2.15E-09 |
| LRRN6A | -4.045 | 2.34E-09 |
| FBXO7 | -5.381 | 2.86E-09 |
| FLJ35934 | -3.471 | 3.00E-09 |
| C18orf1 | -6.220 | 3.89E-09 |
| C3orf37 | -3.077 | 3.95E-09 |
| CCDC48 | -5.971 | 4.24E-09 |
| PLA2G5 | -6.052 | 5.87E-09 |
| SPSB2 | -3.568 | 6.05E-09 |
| KRT36 | -3.443 | 6.43E-09 |
| RGL1 | -5.623 | 6.80E-09 |
| RLN1 | -3.789 | 7.53E-09 |
| B4GALT4 | -3.216 | 7.53E-09 |
| SMUG1 | -3.577 | 8.35E-09 |
| PDIA4 | -6.307 | 1.11E-08 |
| NDUFB10 | -2.827 | 1.11E-08 |
| U1SNRNPBP | -5.560 | 1.12E-08 |
| CEP164 | -6.440 | 1.47E-08 |
| PARP1 | -3.291 | 1.53E-08 |
| SRD5A1 | -5.380 | 1.61E-08 |
| MYCBP2 | -4.248 | 1.61E-08 |
| ELAVL3 | -4.369 | 1.63E-08 |
| EIF2B5 | -5.521 | 1.99E-08 |
| PAICS | -3.396 | 3.81E-08 |
| NSUN5B | -3.735 | 3.98E-08 |
| CDCP1 | -3.039 | 4.63E-08 |
| FAM51A1 | -3.654 | 4.63E-08 |
| MTO1 | -5.948 | 4.92E-08 |
| C21orf6 | -3.310 | 5.67E-08 |
| PPP1R11 | -3.465 | 5.79E-08 |
| C1QA | -4.668 | 6.30E-08 |
| ZC3H3 | -3.401 | 6.65E-08 |
| UNQ2541 | -3.793 | 1.02E-07 |
| ABHD1 | -3.935 | 1.13E-07 |
| AMD1 | -3.246 | 1.13E-07 |
| DCTN6 | -3.105 | 1.14E-07 |
| CTNNB1 | -3.496 | 1.27E-07 |
| BCL6B | -6.384 | 1.50E-07 |
| UCP1 | -3.874 | 1.54E-07 |
| GIMAP8 | -3.059 | 1.55E-07 |
| PDCD5 | -4.546 | 1.69E-07 |
| ZNF187 | -4.243 | 1.69E-07 |
| NFYB | -3.299 | 1.71E-07 |
| HCFC2 | -4.550 | 1.72E-07 |
| ZMYM5 | -3.396 | 1.72E-07 |
| CHPT1 | -3.340 | 1.92E-07 |
| IFIT2 | -2.939 | 1.98E-07 |
| PIAS3 | -4.035 | 2.00E-07 |
| ATP6V1D | -3.735 | 2.05E-07 |
| SALF | -4.344 | 2.35E-07 |
| VAMP5 | -2.697 | 2.81E-07 |
| GLA | -2.361 | 2.83E-07 |
| PVRL3 | -4.453 | 2.87E-07 |
| GRAMD1A | -2.574 | 3.64E-07 |
| MLF1IP | -4.032 | 4.03E-07 |
| KIAA0446 | -3.555 | 4.38E-07 |
| PPIL6 | -4.389 | 4.38E-07 |
| SAC3D1 | -2.573 | 4.87E-07 |
| SLC6A12 | -3.098 | 5.17E-07 |
| ACSL6 | -4.217 | 6.15E-07 |
| OSCAR | -2.744 | 6.52E-07 |
| RPS17 | -2.764 | 7.54E-07 |
| FLJ20294 | -2.993 | 7.70E-07 |
| MTRF1L | -2.804 | 7.71E-07 |
| HAVCR2 | -2.719 | 8.08E-07 |
| DHFRL1 | -5.188 | 8.86E-07 |
| CNFN | -3.233 | 9.12E-07 |
| UBXD6 | -3.414 | 9.22E-07 |
| SPANXA1 | -3.931 | 9.88E-07 |
| ARL4A | -3.489 | 1.10E-06 |
| RNF141 | -2.534 | 1.10E-06 |
| PCDHB6 | -2.741 | 1.10E-06 |
| C1orf170 | -2.300 | 1.21E-06 |
| ANKRD20A2 | -2.267 | 1.23E-06 |
| ASMTL | -4.011 | 1.23E-06 |
| HTR4 | -2.094 | 1.28E-06 |
| C11orf56 | -3.502 | 1.33E-06 |
| FAM49B | -2.107 | 1.33E-06 |
| H3F3B | -3.774 | 1.46E-06 |
| KCNJ4 | -2.719 | 1.46E-06 |
| DMKN | -4.601 | 1.58E-06 |
| BRD9 | -3.415 | 1.60E-06 |
| CDC42SE2 | -3.661 | 1.63E-06 |
| ZNF527 | -2.829 | 1.74E-06 |
| FAP | -3.982 | 1.74E-06 |
| RAB30 | -2.899 | 1.77E-06 |
| KCNJ3 | -3.428 | 1.77E-06 |
| NXF3 | -2.846 | 1.96E-06 |
| RSHL2 | -2.093 | 2.23E-06 |
| SYNJ2BP | -3.479 | 2.29E-06 |
| SCGB2A2 | -3.610 | 2.46E-06 |
| DDEF1 | -2.512 | 2.79E-06 |
| LAMC1 | -2.777 | 2.99E-06 |
| MLL5 | -2.971 | 3.08E-06 |
| ABCB1 | -3.491 | 3.11E-06 |
| HEATR2 | -5.642 | 3.13E-06 |
| RC74 | -2.501 | 3.14E-06 |
| KRT20 | -2.176 | 3.38E-06 |
| INSL3 | -5.793 | 3.43E-06 |
| LTC4S | -3.241 | 3.99E-06 |
| C1orf102 | -2.872 | 4.47E-06 |
| TLR2 | -5.499 | 4.47E-06 |
| SLC13A3 | -2.337 | 4.82E-06 |
| MCF2 | -3.546 | 4.90E-06 |
| DDX54 | -2.443 | 5.18E-06 |
| TAIP-2 | -2.449 | 5.82E-06 |
| B3GNT3 | -2.785 | 5.82E-06 |
| CLCC1 | -2.193 | 6.41E-06 |
| DLD | -3.250 | 6.41E-06 |
| MMP28 | -1.874 | 6.41E-06 |
| BCL11A | -3.889 | 6.54E-06 |
| DIP13B | -2.697 | 6.56E-06 |
| PPP1R2 | -3.427 | 6.58E-06 |
| RNF149 | -2.303 | 6.75E-06 |
| FEV | -1.898 | 6.93E-06 |
| PSCD3 | -2.581 | 7.56E-06 |
| C9orf74 | -2.310 | 7.77E-06 |
| THRAP3 | -2.324 | 8.06E-06 |
| CHN1 | -3.124 | 8.98E-06 |
| P2RXL1 | -3.949 | 9.37E-06 |
| GPR176 | -3.377 | 1.03E-05 |
| SPR | -2.698 | 1.10E-05 |
| KIAA1904 | -2.301 | 1.20E-05 |
| HRASLS | -1.654 | 1.25E-05 |
| USP8 | -4.170 | 1.33E-05 |
| COPS6 | -2.375 | 1.42E-05 |
| HDAC8 | -2.254 | 1.42E-05 |
| CEACAM1 | -2.131 | 1.61E-05 |
| FLJ44186 | -4.345 | 1.62E-05 |
| MAPK15 | -1.695 | 1.68E-05 |
| C4orf13 | -2.103 | 1.81E-05 |
| SPFH2 | -2.394 | 1.82E-05 |
| SLC25A4 | -2.627 | 1.82E-05 |
| DNMT3B | -2.482 | 1.91E-05 |
| MAP3K3 | -2.400 | 1.97E-05 |
| LDOC1 | -3.261 | 1.98E-05 |
| ECD | -1.978 | 2.07E-05 |
| TMEM32 | -3.051 | 2.08E-05 |
| ALS2CR7 | -2.189 | 2.20E-05 |
| KIAA1370 | -2.396 | 2.28E-05 |
| MYLPF | -2.949 | 2.39E-05 |
| LRIG3 | -2.598 | 2.43E-05 |
| MALT1 | -2.586 | 2.43E-05 |
| CLGN | -3.037 | 2.46E-05 |
| BMSC-MCP | -2.972 | 2.56E-05 |
| SLC28A1 | -2.667 | 2.66E-05 |
| INHBC | -2.497 | 2.67E-05 |
| OR10G3 | -1.909 | 2.68E-05 |
| TSC1 | -1.951 | 2.70E-05 |
| DYRK3 | -2.925 | 2.79E-05 |
| C1QTNF3 | -2.088 | 2.95E-05 |
| EIF1B | -2.631 | 3.01E-05 |
| NDUFB9 | -2.467 | 3.15E-05 |
| ATG5 | -2.755 | 3.23E-05 |
| TIMP2 | -2.812 | 3.44E-05 |
| OR2B3 | -3.264 | 3.50E-05 |
| BPY2B | -1.634 | 3.61E-05 |
| ABCF3 | -2.781 | 3.69E-05 |
| CNR2 | -1.834 | 3.88E-05 |
| CBX4 | -1.993 | 3.94E-05 |
| EPO | -2.251 | 4.34E-05 |
| ACRV1 | -2.098 | 4.36E-05 |
| SLC5A8 | -2.995 | 4.70E-05 |
| SCAND1 | -2.996 | 4.98E-05 |
| DHDH | -2.281 | 5.05E-05 |
| TRPV4 | -2.189 | 5.05E-05 |
| MED9 | -2.661 | 5.12E-05 |
| YBX1 | -3.156 | 5.12E-05 |
| ADAR | -2.618 | 5.31E-05 |
| MYCT1 | -2.546 | 5.35E-05 |
| NDUFA10 | -4.782 | 5.93E-05 |
| PCDH19 | -2.424 | 5.97E-05 |
| HPSE | -2.398 | 6.16E-05 |
| MDH1 | -1.762 | 6.37E-05 |
| VMD2L2 | -2.243 | 6.41E-05 |
| KIF18A | -2.956 | 6.41E-05 |
| FAM89B | -2.321 | 6.49E-05 |
| PLD4 | -2.792 | 6.51E-05 |
| PPP3CC | -3.016 | 6.77E-05 |
| TMEFF2 | -1.590 | 7.37E-05 |
| ZNF307 | -2.147 | 7.39E-05 |
| MEGF11 | -1.945 | 7.41E-05 |
| SNAP25 | -3.432 | 7.61E-05 |
| SGPP2 | -2.219 | 7.76E-05 |
| SLC25A36 | -2.144 | 7.87E-05 |
| SPAG5 | -3.483 | 7.98E-05 |
| AMZ2 | -2.463 | 8.04E-05 |
| HSPA14 | -2.712 | 8.04E-05 |
| FBP2 | -3.316 | 8.13E-05 |
| C8orf4 | -2.055 | 8.24E-05 |
| VPS11 | -1.846 | 8.28E-05 |
| C7orf16 | -1.798 | 8.29E-05 |
| ANKRD6 | -3.114 | 8.52E-05 |
| LOC51336 | -2.380 | 8.62E-05 |
| C9orf114 | -2.471 | 8.68E-05 |
| LUZPP1 | -3.247 | 8.80E-05 |
| DHRS1 | -2.619 | 9.26E-05 |
| CASQ1 | -1.603 | 9.46E-05 |
| PDZD7 | -3.471 | 9.67E-05 |
| TTC32 | -3.015 | 9.74E-05 |
| DOCK4 | -3.397 | 9.74E-05 |
| SIAH1 | -1.939 | 9.92E-05 |
| ADAMTS2 | -2.987 | 0.00010035 |
| LOC155060 | -1.771 | 0.00010071 |
| CDKN3 | -3.107 | 0.00011715 |
| CARHSP1 | -2.194 | 0.00011937 |
| ZNF646 | -3.227 | 0.00011937 |
| ARF1 | -2.899 | 0.00012085 |
| OR4A47 | -3.804 | 0.00012085 |
| EIF5A | -1.987 | 0.00012268 |
| MSH3 | -2.311 | 0.00012316 |
| CYP27B1 | -2.380 | 0.00012412 |
| RECQL5 | -4.217 | 0.00012502 |
| ST6GALNAC2 | -2.861 | 0.00012633 |
| TMEM9B | -2.619 | 0.00012756 |
| UGT8 | -1.543 | 0.00012808 |
| FBXO18 | -2.338 | 0.0001295 |
| CNGA1 | -2.656 | 0.00012973 |
| C15orf39 | -2.036 | 0.00013162 |
| SMARCA5 | -2.697 | 0.00013162 |
| TNFSF5IP1 | -2.620 | 0.00013764 |
| DNAJA5 | -2.615 | 0.00013849 |
| RSBN1 | 2.720 | 0.00013898 |
| HSPC048 | -2.506 | 0.00014409 |
| C17orf71 | -3.093 | 0.00014496 |
| MOBKL2A | -1.967 | 0.00014776 |
| AVPR1A | -2.102 | 0.00014828 |
| BTAF1 | -2.153 | 0.00015677 |
| TMED10 | -1.822 | 0.00015677 |
| MGC22793 | -1.811 | 0.00016591 |
| TLK1 | -3.269 | 0.00016591 |
| ZNF26 | -2.929 | 0.00018195 |
| ABCA8 | -2.158 | 0.00018446 |
| MEGF10 | -2.588 | 0.00019533 |
| C20orf173 | -1.790 | 0.00020504 |
| SP100 | -1.605 | 0.00020504 |
| HIST1H2AL | -2.848 | 0.00020508 |
| PITX1 | -2.814 | 0.00020814 |
| FEM1C | -2.331 | 0.00020814 |
| CYP11A1 | -1.910 | 0.00021035 |
| GINS4 | -1.661 | 0.00021035 |
| LRP10 | -2.105 | 0.00021035 |
| CCND3 | -2.448 | 0.00021371 |
| C7orf10 | -2.271 | 0.00021461 |
| CCNJ | -2.724 | 0.000216 |
| HMBOX1 | -2.719 | 0.00021657 |
| FLJ21438 | -2.270 | 0.00021808 |
| C19orf24 | -2.625 | 0.0002233 |
| RDX | -1.764 | 0.00022947 |
| TRUB1 | -2.724 | 0.00023152 |
| FOXI1 | -1.580 | 0.00024152 |
| EDG1 | -1.564 | 0.00026605 |
| OR4K14 | -2.743 | 0.00026605 |
| PDCD2 | -2.125 | 0.00026605 |
| GPATC4 | -3.149 | 0.00028079 |
| KATNAL1 | -1.829 | 0.00028079 |
| NUDT4 | -2.736 | 0.00029794 |
| IK | -2.325 | 0.00029928 |
| AKAP11 | -1.752 | 0.00029928 |
| C6orf21 | -1.860 | 0.00029928 |
| CRYBB1 | -1.668 | 0.00029928 |
| DPH2 | -2.063 | 0.00029928 |
| GTF2A2 | -2.533 | 0.00029928 |
| KCNIP2 | -1.985 | 0.00029928 |
| NUPR1 | -2.445 | 0.00029928 |
| RPA1 | -1.927 | 0.00029928 |
| USP32 | -3.517 | 0.00029928 |
| PRDM10 | -1.312 | 0.00030148 |
| CD300C | -2.597 | 0.00030963 |
| C12orf49 | -1.412 | 0.00032894 |
| ISOC1 | -2.852 | 0.00033406 |
| CD99L2 | -2.449 | 0.00033523 |
| TFF3 | -1.770 | 0.00034616 |
| C15orf48 | -2.877 | 0.00034633 |
| OR51L1 | -2.629 | 0.00034816 |
| DHRS9 | -5.112 | 0.00034816 |
| LPL | -1.866 | 0.00034816 |
| NRBP1 | -2.647 | 0.00034896 |
| DCK | -3.060 | 0.0003629 |
| HLA-DPA1 | -2.182 | 0.00036605 |
| ILVBL | -3.203 | 0.00036605 |
| NEUROG3 | -2.982 | 0.00036605 |
| RNASEH1 | -2.822 | 0.00037948 |
| BIRC5 | -3.579 | 0.00038458 |
| PLCB1 | -1.227 | 0.00038653 |
| CFTR | -2.577 | 0.00038977 |
| ARL13A | -2.498 | 0.00039064 |
| CHST5 | -2.262 | 0.00039064 |
| NNT | -1.470 | 0.00039635 |
| BAIAP2L1 | -2.163 | 0.00040348 |
| NAV1 | -1.732 | 0.00040902 |
| COX5B | -1.987 | 0.00040935 |
| IL2RB | -2.469 | 0.00041115 |
| LRPPRC | -3.199 | 0.00042168 |
| C14orf106 | -1.796 | 0.00042889 |
| CDX4 | -2.171 | 0.00042933 |
| ZNF507 | -2.164 | 0.00042939 |
| DBI | -1.668 | 0.00043501 |
| RACGAP1P | -2.068 | 0.00043684 |
| FLJ20366 | -2.422 | 0.00045429 |
| CHM | -1.949 | 0.00045507 |
| KYNU | -1.922 | 0.00045591 |
| KLHL25 | -2.374 | 0.0004831 |
| RAB17 | -1.708 | 0.0004831 |
| RP11-255A11.18 | -2.803 | 0.0005019 |
| IDI2 | -2.108 | 0.00050621 |
| S100A5 | -2.088 | 0.00050621 |
| FPRL2 | -4.439 | 0.00050763 |
| RNF111 | -1.755 | 0.00051483 |
| PRB1 | -1.303 | 0.00051649 |
| ADCY2 | -1.653 | 0.0005191 |
| SRCRB4D | -2.198 | 0.00054381 |
| C13orf7 | -1.591 | 0.00055176 |
| ANKRD7 | -2.404 | 0.00056757 |
| TTC22 | -2.717 | 0.00057189 |
| KIFC3 | -2.250 | 0.00058148 |
| C3orf14 | -2.427 | 0.00062349 |
| TRIM6 | 1.721 | 0.00062349 |
| MRPL3 | -1.454 | 0.00062581 |
| RFWD2 | -1.863 | 0.00063538 |
| BHMT | -2.664 | 0.00063783 |
| RNPEP | -4.498 | 0.00063783 |
| ARL2BP | -3.054 | 0.00064146 |
| ITIH3 | -1.557 | 0.00064146 |
| ACTR5 | -1.781 | 0.00064517 |
| ALPL | -2.542 | 0.00065822 |
| C9orf125 | -2.386 | 0.00066043 |
| SEC24C | -2.915 | 0.00066405 |
| ATG12 | -1.553 | 0.00066771 |
| PSCD2 | -2.244 | 0.00066795 |
| TTC26 | -2.144 | 0.00067561 |
| ANK2 | -1.598 | 0.0006889 |
| SNFT | -2.130 | 0.00071467 |
| ARV1 | -1.502 | 0.00072438 |
| C11orf58 | -1.206 | 0.00072438 |
| IDE | -2.178 | 0.0007564 |
| TALDO1 | -2.356 | 0.00077534 |
| SLC39A10 | -1.697 | 0.00080036 |
| PRO1853 | -1.864 | 0.00081335 |
| CAPNS2 | -1.327 | 0.00083536 |
| BIRC2 | -1.945 | 0.00085375 |
| MMP23B | -2.131 | 0.00086316 |
| ZNF606 | -2.033 | 0.00086316 |
| DKFZp686I1569 | -2.177 | 0.00091603 |
| MCOLN3 | -1.466 | 0.00091689 |
| TMC3 | -1.749 | 0.0009323 |
| RP6-213H19.1 | -2.149 | 0.00094064 |
| DIDO1 | -6.053 | 0.000945 |
| TFDP2 | -2.006 | 0.00095742 |
| KIAA1411 | -2.580 | 0.00100032 |
| CAMK2A | -1.697 | 0.00100661 |
| TXNDC11 | -1.744 | 0.00103238 |
| ZNF157 | -1.975 | 0.00103238 |
| CRYBB2 | -1.684 | 0.00104297 |
| MEFV | -1.941 | 0.00106816 |
| OLFM1 | -1.677 | 0.00106956 |
| GINS1 | -2.201 | 0.00111189 |
| NOX3 | -1.656 | 0.00111189 |
| FOXP2 | -2.274 | 0.00111469 |
| SLC39A14 | -2.283 | 0.00111516 |
| LIN7C | -1.675 | 0.00112078 |
| SPAG17 | -2.093 | 0.00112078 |
| TMEM79 | 2.223 | 0.00112078 |
| APOA1BP | -2.037 | 0.00112877 |
| APOB48R | -2.365 | 0.00112877 |
| TMEM127 | -3.054 | 0.00112877 |
| NPR2 | -1.443 | 0.00114813 |
| PPP2R5C | -1.764 | 0.00115486 |
| KLHL1 | -1.780 | 0.00117673 |
| OMG | -1.533 | 0.00117876 |
| ZNF443 | -1.126 | 0.00117876 |
| ZNF416 | -1.779 | 0.00119102 |
| KIR2DL5A | -1.817 | 0.00121018 |
| NRSN1 | -2.220 | 0.00121018 |
| GMPS | -2.056 | 0.00123154 |
| NALP7 | -1.839 | 0.00123154 |
| TM4SF4 | -2.910 | 0.00123154 |
| PRPF6 | -3.141 | 0.00123668 |
| DAPK2 | -2.005 | 0.00124774 |
| HLA-G | -2.140 | 0.00124774 |
| KCNG1 | -2.749 | 0.00124774 |
| MGC4677 | -2.854 | 0.00124774 |
| PLEKHN1 | -2.071 | 0.00124915 |
| KIF1B | -1.944 | 0.0013207 |
| CDC14A | -1.831 | 0.00133872 |
| TMEM119 | -2.348 | 0.00134366 |
| KIAA1772 | -2.983 | 0.00137015 |
| RPS27A | -1.699 | 0.0014038 |
| CROT | -1.922 | 0.00141367 |
| KRTAP5-9 | -1.282 | 0.00141649 |
| KRTAP9-3 | -2.307 | 0.00141649 |
| MAP3K14 | -2.232 | 0.00141997 |
| EIF5A2 | -1.878 | 0.00144487 |
| SPAG16 | 2.037 | 0.00145726 |
| ARL6IP | -1.325 | 0.00149871 |
| PTTG1 | -1.517 | 0.00149871 |
| CSTL1 | -2.256 | 0.00151421 |
| C6orf10 | -1.510 | 0.00152055 |
| BTBD15 | -1.976 | 0.00154389 |
| MYH3 | -2.405 | 0.00154389 |
| PPM1E | -1.921 | 0.00154389 |
| ADAM17 | -2.144 | 0.00156728 |
| PDYN | -2.641 | 0.00159417 |
| SCAND2 | -1.560 | 0.00161083 |
| C14orf8 | -1.865 | 0.00163709 |
| ZNF548 | -3.271 | 0.00164921 |
| PFAAP5 | -3.119 | 0.00169537 |
| KBTBD5 | -2.246 | 0.00171441 |
| C11orf41 | -1.914 | 0.00171912 |
| SERTAD3 | -1.988 | 0.00174094 |
| NAP1L4 | -1.565 | 0.00176668 |
| PHYHD1 | -1.769 | 0.00178102 |
| PHF17 | -1.589 | 0.00178224 |
| PINK1 | -1.705 | 0.00182928 |
| ATXN7 | -1.959 | 0.00183588 |
| MAWBP | 1.859 | 0.00185479 |
| NEIL1 | -1.140 | 0.00185479 |
| MUC20 | -1.469 | 0.00186413 |
| ROGDI | -2.222 | 0.00186956 |
| ZNF175 | -2.356 | 0.00187495 |
| MYH2 | -3.845 | 0.00190473 |
| GLS2 | -3.084 | 0.00204305 |
| MMP14 | -1.807 | 0.00205017 |
| CAPN11 | -2.076 | 0.00209137 |
| ALKBH5 | -1.912 | 0.00209749 |
| C11orf49 | -1.764 | 0.00210068 |
| ENSA | -1.568 | 0.00210068 |
| OLFM3 | -1.725 | 0.00210434 |
| CLPTM1 | 1.960 | 0.00216112 |
| UNC13B | -1.061 | 0.00219748 |
| PMFBP1 | -1.449 | 0.00220956 |
| CBLL1 | -1.933 | 0.00221671 |
| TRAK2 | -1.378 | 0.00229807 |
| GLRX | -1.332 | 0.00231283 |
| TRIT1 | -1.592 | 0.00238234 |
| TPRKB | -2.167 | 0.00239425 |
| HS3ST2 | -2.980 | 0.00240507 |
| C14orf153 | -1.160 | 0.0024106 |
| MAPK3 | -4.171 | 0.00241734 |
| GPR85 | -1.918 | 0.00243586 |
| CHST7 | -1.655 | 0.00245797 |
| C21orf42 | -1.837 | 0.00252782 |
| FAM112B | -2.986 | 0.00255159 |
| E2F6 | -1.768 | 0.00260428 |
| RFXDC2 | -1.421 | 0.00260428 |
| TCEAL5 | -3.395 | 0.00260428 |
| C4orf16 | -2.558 | 0.0026419 |
| IGF1R | -1.424 | 0.00267509 |
| CXCL16 | -1.558 | 0.00273483 |
| HLXB9 | -1.374 | 0.00273483 |
| KRT17 | -2.420 | 0.00273483 |
| OR4N2 | -1.589 | 0.00274726 |
| ALDH1A1 | -2.585 | 0.00277652 |
| CTDSPL | -2.567 | 0.00279696 |
| NOTUM | -1.558 | 0.00279696 |
| OCIAD1 | -1.653 | 0.00286485 |
| RAB3D | -2.054 | 0.0028841 |
| PAFAH1B3 | -2.175 | 0.00290041 |
| DMWD | -1.391 | 0.0029185 |
| SDHA | -1.789 | 0.00295723 |
| ANGPT4 | -3.271 | 0.00304083 |
| ODF1 | -1.254 | 0.00307035 |
| DUSP2 | -2.193 | 0.00311451 |
| DUSP15 | -0.937 | 0.00315135 |
| NMNAT3 | -1.902 | 0.00321103 |
| TFAP4 | -1.981 | 0.00323474 |
| DDX24 | 1.740 | 0.00330433 |
| DKFZp686L1814 | -2.380 | 0.00330433 |
| GBP4 | -1.966 | 0.00331259 |
| PCYT2 | -1.574 | 0.00331259 |
| CD28 | 2.438 | 0.00333857 |
| CCPG1 | 1.036 | 0.00346365 |
| METRN | -1.892 | 0.00370487 |
| PITPNM1 | -1.175 | 0.00381145 |
| SEMA6A | -1.086 | 0.00381328 |
| NYREN18 | 1.353 | 0.0038459 |
| IPF1 | -1.247 | 0.00387028 |
| EML2 | -1.569 | 0.00387246 |
| CDC16 | -1.838 | 0.00390874 |
| STARD7 | -1.774 | 0.00397621 |
| PCYT1A | -1.881 | 0.00402473 |
| DTYMK | -2.353 | 0.00403742 |
| CGRRF1 | -1.707 | 0.00403742 |
| BCORL1 | -1.475 | 0.00405893 |
| FGG | -1.531 | 0.00405893 |
| SAFB | -1.175 | 0.00405893 |
| CCRK | -1.749 | 0.00406246 |
| HIST1H3C | -1.970 | 0.00407367 |
| MLNR | -1.112 | 0.00409295 |
| ABCG1 | 1.663 | 0.00417562 |
| MAPK12 | -1.857 | 0.00417562 |
| OTOR | -2.103 | 0.00417562 |
| SOX4 | -1.313 | 0.00417562 |
| TEGT | -2.003 | 0.00419913 |
| CA14 | -1.344 | 0.00420332 |
| CENPL | -1.713 | 0.00425487 |
| LHFP | -2.039 | 0.00425487 |
| LIN7B | -1.493 | 0.00425487 |
| STEAP3 | -1.588 | 0.00425487 |
| TTC7B | -2.248 | 0.00425487 |
| ZNF563 | -2.131 | 0.0043152 |
| CDH26 | -1.688 | 0.00436719 |
| COQ6 | -1.606 | 0.00442417 |
| MTMR9 | -1.286 | 0.00456895 |
| KCNAB1 | -2.752 | 0.00458851 |
| CYP2W1 | -1.552 | 0.00464057 |
| ZHX3 | -1.380 | 0.00464057 |
| TMBIM4 | -2.273 | 0.00472617 |
| TERF2IP | -1.549 | 0.00472726 |
| MARCKS | -1.858 | 0.00472726 |
| PDE4C | -2.046 | 0.00472726 |
| RNF10 | 1.892 | 0.00476305 |
| MEGF6 | -1.436 | 0.00483101 |
| KIAA0258 | -1.431 | 0.00491542 |
| C21orf58 | -2.513 | 0.00493151 |
| KCNK7 | -1.064 | 0.00493279 |
| LRRN3 | -1.237 | 0.00493279 |
| ZNF746 | -1.602 | 0.00503179 |
| MTMR10 | -1.342 | 0.00512249 |
| C21orf77 | -1.495 | 0.0051434 |
| IL11 | -2.653 | 0.00514495 |
| RP5-821D11.2 | -2.579 | 0.00517324 |
| SLC43A1 | -2.027 | 0.00521525 |
| OR5AK2 | -1.955 | 0.00522453 |
| LRRN5 | 2.274 | 0.00523377 |
| PAGE5 | -1.671 | 0.00525362 |
| ZBED3 | -2.155 | 0.00530715 |
| PHKA2 | 2.552 | 0.00539059 |
| PLEKHA4 | -1.684 | 0.00539059 |
| RAB3IP | -2.403 | 0.00539059 |
| BTBD1 | -2.208 | 0.00543831 |
| PRPF40A | -1.863 | 0.00544241 |
| HPSE2 | -1.117 | 0.00550818 |
| C1orf129 | 1.707 | 0.00563097 |
| KNG1 | -1.739 | 0.0056559 |
| ADAT1 | -1.557 | 0.0056701 |
| ADMR | -1.551 | 0.0056701 |
| KLK4 | -1.479 | 0.00567813 |
| TCP11 | 2.166 | 0.00573559 |
| FBXO5 | -2.006 | 0.00573559 |
| LOC285148 | -1.656 | 0.00573559 |
| OSTalpha | 1.478 | 0.00573559 |
| FAS | -1.301 | 0.00588277 |
| DNAJB4 | -1.995 | 0.00591481 |
| MAGEE2 | 1.463 | 0.00591481 |
| C12orf24 | -1.851 | 0.0059488 |
| PSMA8 | 1.928 | 0.00598689 |
| LOC646627 | -1.644 | 0.00599142 |
| C16orf61 | -1.317 | 0.00599914 |
| HOXD3 | -2.037 | 0.00603183 |
| CPB1 | -2.008 | 0.00603183 |
| RAB35 | -1.034 | 0.00603183 |
| HMP19 | -1.343 | 0.00603908 |
| C11orf60 | -1.675 | 0.00608398 |
| COMMD8 | -1.248 | 0.00621541 |
| SLC34A2 | -1.785 | 0.00629285 |
| ANGPTL4 | -1.242 | 0.00629377 |
| TRAM1 | -1.249 | 0.00629377 |
| EIF3S6IP | -1.770 | 0.0063057 |
| RAPGEF4 | -2.184 | 0.00639398 |
| KRT81 | -1.484 | 0.00642341 |
| RAB20 | -1.780 | 0.00645391 |
| BRPF1 | -1.640 | 0.00650711 |
| KCND3 | -1.324 | 0.00653991 |
| SIPA1L1 | -1.699 | 0.00659417 |
| ACCN3 | -1.296 | 0.00661194 |
| PANK1 | -1.217 | 0.00663596 |
| ICAM3 | -1.759 | 0.00665831 |
| KIAA1018 | -0.949 | 0.00666329 |
| GP2 | -2.330 | 0.00673645 |
| LBX2 | -1.106 | 0.00673645 |
| CLK2 | -2.461 | 0.00678589 |
| C22orf5 | -1.078 | 0.00680847 |
| XRCC6BP1 | -1.900 | 0.00685553 |
| KIAA0907 | -1.258 | 0.00693771 |
| ZRANB3 | -0.959 | 0.00695311 |
| DNAJB6 | -2.159 | 0.00696902 |
| CCL17 | -2.352 | 0.00699767 |
| TFPT | -1.490 | 0.00699767 |
| TRAF6 | 1.855 | 0.00704724 |
| GPC5 | -1.641 | 0.00705974 |
| TIGD5 | -1.242 | 0.00708017 |
| CXorf2 | -1.686 | 0.00711989 |
| ZNF395 | -1.245 | 0.00711989 |
| ZCD1 | -1.809 | 0.00717138 |
| FIP1L1 | -1.742 | 0.00717138 |
| PLCG1 | -1.779 | 0.00717138 |
| ZNF213 | -2.864 | 0.00719978 |
| C11orf68 | -1.812 | 0.00728368 |
| RWDD3 | -1.562 | 0.00728368 |
| RAB9A | 2.892 | 0.00730192 |
| DKFZP586H2123 | -1.451 | 0.00738521 |
| CXorf6 | -2.100 | 0.00747536 |
| NYD-SP26 | -1.989 | 0.00772766 |
| EIF2B2 | -1.568 | 0.00772766 |
| MLLT3 | -1.527 | 0.00772766 |
| EIF4ENIF1 | -1.668 | 0.00776571 |
| IQCA | -1.178 | 0.00776571 |
| ATF5 | -1.361 | 0.00776768 |
| EPHA4 | -2.197 | 0.00783915 |
| DECR1 | 3.009 | 0.00797213 |
| KCNE2 | 1.571 | 0.00797213 |
| RPS11 | -1.458 | 0.00797301 |
| SLC26A2 | -3.365 | 0.0081065 |
| STAR | -1.764 | 0.00812697 |
| MTHFSD | -1.412 | 0.00827509 |
| YIF1A | -1.651 | 0.00854183 |
| CHD8 | -1.811 | 0.00866757 |
| TGIF2 | 2.525 | 0.00866757 |
| PDZD3 | -1.917 | 0.00876485 |
| LIN28 | 3.186 | 0.00878755 |
| LACTB | -2.411 | 0.00883929 |
| PEX13 | -1.300 | 0.00893857 |
| PCDHB10 | -1.066 | 0.00902612 |
| CAMK4 | 1.584 | 0.00907806 |
| RGS10 | -1.359 | 0.00916392 |
| PDE3A | -1.191 | 0.00916392 |
| LSM2 | -1.152 | 0.00931236 |
| RAB6IP2 | -1.421 | 0.00931236 |
| NSUN6 | -1.351 | 0.00932693 |
| SENP1 | -1.389 | 0.00932693 |
| SPIN-2 | -1.300 | 0.00936398 |
| CEP76 | -1.454 | 0.00942348 |
| TRIM2 | -1.166 | 0.00945461 |
| KIAA1908 | -1.330 | 0.00949868 |
| WISP2 | -2.062 | 0.0096113 |
| SFRS11 | -1.244 | 0.00962761 |
| CD3EAP | -2.084 | 0.00971025 |
| KIAA1160 | -1.517 | 0.00972856 |
| OPLAH | -1.202 | 0.00989249 |
| PRPH | -1.828 | 0.00989249 |
| GTF2H4 | -1.630 | 0.00992471 |
| CDC2L1 | -1.650 | 0.00996275 |
| TM7SF3 | -1.472 | 0.0100683 |
| ARHGEF6 | -1.597 | 0.01007549 |
| REG1A | -1.431 | 0.01007894 |
| SDC1 | -1.337 | 0.01011152 |
| SEPHS2 | -2.432 | 0.01012573 |
| GCSH | -2.204 | 0.01012573 |
| CAMLG | -1.913 | 0.01014338 |
| PRDX6 | 1.770 | 0.01020595 |
| HLA-A | -1.232 | 0.01038546 |
| PNN | -1.701 | 0.01038546 |
| ACAA2 | -1.428 | 0.01048289 |
| RND1 | 1.400 | 0.01052427 |
| TAZ | -1.666 | 0.01052427 |
| MME | -1.166 | 0.01060888 |
| PACSIN3 | -1.379 | 0.01060888 |
| PRM1 | 1.717 | 0.01061723 |
| BNC1 | -2.792 | 0.01078101 |
| PREB | -1.304 | 0.0107852 |
| RP11-298P3.3 | -1.838 | 0.01087537 |
| GLTSCR2 | -2.030 | 0.01087537 |
| STIM1 | -2.192 | 0.01087537 |
| HOXD10 | -1.045 | 0.01102918 |
| GRB14 | -0.868 | 0.01134411 |
| BPI | -1.531 | 0.0113799 |
| KATNB1 | -1.372 | 0.0113799 |
| EHMT2 | -1.259 | 0.01179896 |
| IGSF4 | -2.551 | 0.01180056 |
| CHRDL2 | -1.012 | 0.01193217 |
| MGC11102 | -1.031 | 0.01194213 |
| LOC497190 | -1.210 | 0.01200709 |
| BRIP1 | -1.532 | 0.01203165 |
| G3BP2 | -1.719 | 0.01203165 |
| ST3GAL4 | -1.057 | 0.01211032 |
| ZNF208 | -0.952 | 0.0121533 |
| OTEX | -1.503 | 0.01222442 |
| EAF2 | -1.735 | 0.012484 |
| HAGH | 2.313 | 0.012484 |
| PLA2G7 | -1.442 | 0.012484 |
| VASP | -1.405 | 0.01249749 |
| AOF2 | -1.589 | 0.01257522 |
| ALKBH6 | -1.794 | 0.01258875 |
| GNAT1 | -2.346 | 0.01278825 |
| SYT8 | -2.048 | 0.01288641 |
| HIST1H3D | -1.876 | 0.01298654 |
| PKHD1L1 | -1.424 | 0.01304841 |
| POU2F1 | 2.110 | 0.01306201 |
| RCCD1 | -1.617 | 0.01317464 |
| SH3BP4 | -1.594 | 0.01322317 |
| C1QTNF9 | -1.361 | 0.01324146 |
| DHCR7 | -1.675 | 0.01324146 |
| ELL3 | 2.620 | 0.01326786 |
| TMEM139 | -1.196 | 0.01329764 |
| ZFYVE16 | -2.349 | 0.01349555 |
| ARC | -1.968 | 0.01360827 |
| DPP7 | -1.092 | 0.01360827 |
| PLXNB3 | -1.732 | 0.01360827 |
| FKBP8 | -1.562 | 0.01425883 |
| UNQ1887 | -1.917 | 0.01434457 |
| ACAA1 | -1.056 | 0.014448 |
| POGZ | 2.146 | 0.014448 |
| MS4A7 | -1.446 | 0.01448549 |
| ZNF121 | -1.320 | 0.0145474 |
| CNN1 | -1.178 | 0.01466267 |
| ZNF433 | -1.157 | 0.01474405 |
| CXCL6 | -1.091 | 0.01475221 |
| ARNT2 | -1.484 | 0.01479728 |
| GAS8 | -2.475 | 0.01479728 |
| TM9SF2 | -1.381 | 0.01497498 |
| LRRC36 | -1.872 | 0.01499626 |
| KIAA1900 | -1.094 | 0.01523192 |
| ZNF415 | -1.187 | 0.01523648 |
| MIER2 | -1.396 | 0.01524662 |
| KPNA5 | -2.120 | 0.01528215 |
| MORC1 | -1.472 | 0.01532767 |
| ADIPOR2 | -1.441 | 0.01542404 |
| LEMD3 | -1.362 | 0.0155715 |
| COMMD3 | -1.453 | 0.01569005 |
| GATA5 | -1.614 | 0.01569005 |
| CENPK | -1.561 | 0.01579938 |
| CSDA | 1.554 | 0.01605976 |
| GPS2 | -1.396 | 0.01619084 |
| OPN4 | 2.134 | 0.01619084 |
| EFHD2 | -1.741 | 0.01619518 |
| VEGFC | -1.363 | 0.01639593 |
| CCNA1 | -1.939 | 0.01661848 |
| HS2ST1 | -2.055 | 0.01662603 |
| IBRDC2 | -1.809 | 0.01668064 |
| HECTD1 | -1.628 | 0.01696518 |
| SP1 | -1.734 | 0.01712738 |
| C6orf35 | -1.374 | 0.01748268 |
| EDEM3 | -1.252 | 0.0174966 |
| ARHGAP17 | 2.128 | 0.01750637 |
| FLT3 | -0.977 | 0.01750637 |
| GPR126 | -1.629 | 0.01756084 |
| MYO9A | 1.548 | 0.01762341 |
| FLJ11996 | -1.561 | 0.01781411 |
| DDX58 | -1.240 | 0.01782064 |
| TLR3 | -0.965 | 0.01782064 |
| CDS1 | -1.141 | 0.01794857 |
| DKFZp762I137 | -1.521 | 0.01797106 |
| CTSW | -1.119 | 0.0179748 |
| PAP2D | -0.994 | 0.01819653 |
| PAX1 | -0.865 | 0.01819653 |
| AP3B2 | -1.234 | 0.01837864 |
| C9orf38 | -0.956 | 0.01837864 |
| FLJ11506 | 2.844 | 0.01847219 |
| FABP7 | -1.681 | 0.01849619 |
| MRPL10 | -1.294 | 0.01849619 |
| PSME3 | 1.685 | 0.01849619 |
| RAP1B | -1.413 | 0.01863484 |
| LAMP1 | -4.357 | 0.01867311 |
| ATPBD1C | -1.345 | 0.01868289 |
| TBC1D16 | -2.137 | 0.01868434 |
| IL11RA | -1.898 | 0.01878406 |
| UBN1 | -1.129 | 0.01878406 |
| CESK1 | 2.727 | 0.01903768 |
| CHI3L2 | 1.591 | 0.01903768 |
| KCNK13 | 3.119 | 0.01933121 |
| MYL6B | -1.699 | 0.01954989 |
| SLC26A11 | -1.639 | 0.01961796 |
| DUSP21 | -1.623 | 0.01970613 |
| CYP21A2 | -0.966 | 0.01984167 |
| HERC5 | -2.109 | 0.01991509 |
| ERCC5 | 2.279 | 0.01994199 |
| TXNL4A | -1.473 | 0.02002777 |
| ASB5 | -1.406 | 0.0201472 |
| REEP2 | -1.697 | 0.0202866 |
| SLC25A5 | -1.269 | 0.0202866 |
| SNRPN | 1.833 | 0.02041472 |
| PCDHB2 | -0.941 | 0.02049342 |
| DNAJC15 | 1.111 | 0.02049342 |
| CTSB | 2.092 | 0.02049342 |
| OR6V1 | -3.027 | 0.02053345 |
| CCDC44 | -1.621 | 0.02073176 |
| SV2A | -1.048 | 0.02078218 |
| UCN2 | -1.011 | 0.02078218 |
| HOXA3 | -1.564 | 0.02078466 |
| CLIC2 | -0.872 | 0.02083948 |
| PFDN4 | 1.412 | 0.02083948 |
| SPRR2B | -1.622 | 0.02083948 |
| RABL4 | -1.396 | 0.020961 |
| CDV3 | -1.593 | 0.02101905 |
| PNLIPRP1 | -1.283 | 0.02101905 |
| PRAP1 | -1.219 | 0.02109343 |
| STRN4 | -1.407 | 0.02110671 |
| C20orf19 | -1.256 | 0.02127932 |
| HADHA | -1.967 | 0.02127932 |
| KRT25 | -1.861 | 0.02127932 |
| PIN1L | -1.886 | 0.02127932 |
| PTP4A1 | -1.820 | 0.02127932 |
| FBXL4 | -1.149 | 0.02149114 |
| CHSY1 | -1.279 | 0.02166315 |
| TMCO3 | 1.546 | 0.02189615 |
| CHST11 | -1.175 | 0.02196696 |
| CYP26A1 | 1.185 | 0.02221712 |
| CAPN14 | -1.278 | 0.02243811 |
| DKFZp434N062 | -1.052 | 0.02243811 |
| TMPRSS4 | -1.518 | 0.02243811 |
| BXDC2 | 2.052 | 0.02260543 |
| DQX1 | -1.993 | 0.02271682 |
| OTUD4 | -1.101 | 0.02279016 |
| TRIM46 | 1.224 | 0.02279016 |
| FRS3 | 1.605 | 0.02290604 |
| IGSF6 | -1.343 | 0.02290604 |
| PNOC | -1.395 | 0.02294514 |
| ATP6V1C1 | -1.485 | 0.02310609 |
| KRTAP1-3 | -1.603 | 0.02310609 |
| SH3TC1 | -1.142 | 0.0233945 |
| PLD3 | 0.758 | 0.02345948 |
| CDY2A | -2.516 | 0.02365339 |
| MFSD1 | -1.292 | 0.02365339 |
| NFIA | -1.342 | 0.02365339 |
| STRC | -1.228 | 0.02372221 |
| MGC10981 | -1.716 | 0.02374813 |
| PTPDC1 | -0.994 | 0.02391988 |
| MDC1 | -1.139 | 0.02410283 |
| OR6B2 | -1.520 | 0.02417884 |
| TRIM23 | -0.820 | 0.02462331 |
| APOL1 | 2.060 | 0.0246978 |
| ERN2 | -1.159 | 0.02473792 |
| NFAM1 | -0.811 | 0.02485793 |
| PFKP | -1.339 | 0.02501111 |
| MRPS2 | -1.211 | 0.02504291 |
| CAMKV | -1.496 | 0.02508443 |
| SAMD4B | -0.783 | 0.02523005 |
| PTPLAD2 | -1.393 | 0.02543016 |
| HSPA5 | -1.495 | 0.02549212 |
| CABP5 | 1.645 | 0.02550344 |
| WDR54 | -1.047 | 0.02550803 |
| BMP15 | -1.331 | 0.0255839 |
| MYO7A | -1.568 | 0.02570253 |
| OR13C4 | -1.751 | 0.02581765 |
| SCML2 | 1.311 | 0.02598003 |
| APXL2 | -1.670 | 0.02598036 |
| C12orf22 | -1.612 | 0.02598036 |
| ERG | -1.269 | 0.02598036 |
| POLQ | -1.526 | 0.02620609 |
| SRGAP3 | 2.184 | 0.02639708 |
| ZNF222 | -1.448 | 0.02640997 |
| DCP1A | -0.842 | 0.02654498 |
| EHD4 | -1.098 | 0.0265486 |
| GTPBP5 | -1.195 | 0.0265486 |
| CTSZ | 0.950 | 0.02660221 |
| GBX2 | 1.548 | 0.0267537 |
| TF | -1.396 | 0.02679967 |
| TCF21 | -1.061 | 0.02685556 |
| WDR45L | -1.664 | 0.02717715 |
| NFKB1 | -1.126 | 0.0273574 |
| CINP | -1.585 | 0.02736687 |
| TATDN3 | -1.143 | 0.02736687 |
| U2AF2 | -1.544 | 0.02747335 |
| YPEL5 | -1.698 | 0.02759684 |
| TRAP1 | -0.947 | 0.02766751 |
| TTLL5 | -1.785 | 0.02808181 |
| CASC5 | 0.769 | 0.02855628 |
| TRIM49 | -1.124 | 0.02872483 |
| TEC | -1.085 | 0.02919227 |
| DNAJC10 | -0.807 | 0.02945168 |
| FASTK | 1.772 | 0.0294616 |
| GALNT2 | -1.603 | 0.0294616 |
| SLC25A12 | 1.530 | 0.02948564 |
| MAST2 | -1.049 | 0.02953084 |
| GYG2 | 1.491 | 0.02953084 |
| RAC2 | 1.985 | 0.02978148 |
| COQ7 | -1.828 | 0.02978836 |
| ATP6AP2 | 1.389 | 0.02989793 |
| DHX32 | -1.059 | 0.02989793 |
| GPR171 | -1.330 | 0.03010866 |
| LRAT | -1.528 | 0.03037554 |
| KCNMA1 | -1.500 | 0.03048033 |
| ZNF688 | 1.226 | 0.03048033 |
| NRL | -1.504 | 0.030526 |
| CENTG3 | 1.350 | 0.03065728 |
| MRPL20 | -2.088 | 0.03065728 |
| FLJ43374 | -1.283 | 0.030756 |
| H6PD | -1.235 | 0.03113237 |
| PDGFC | -1.534 | 0.03113237 |
| CDYL | -1.259 | 0.03116792 |
| DHX8 | -1.098 | 0.03125501 |
| NY-SAR-48 | -1.117 | 0.03139886 |
| DGKI | -1.191 | 0.03150262 |
| SAGE1 | -1.093 | 0.03152571 |
| KIAA2002 | 1.926 | 0.03165004 |
| SLC35D2 | 0.776 | 0.03169139 |
| ANGPT1 | -1.128 | 0.03177947 |
| CIAPIN1 | -1.206 | 0.03184165 |
| COX6A1 | 1.685 | 0.03186673 |
| ATF7 | -1.306 | 0.03206422 |
| DGCR6 | -1.161 | 0.0321657 |
| CACNA1D | -2.042 | 0.03228564 |
| GYPE | 1.075 | 0.03229343 |
| KLF2 | -1.362 | 0.03229343 |
| PAPPA2 | -2.844 | 0.03229343 |
| DMXL2 | 1.751 | 0.03273856 |
| TOR1AIP1 | -1.588 | 0.03284852 |
| GAA | -1.726 | 0.03304746 |
| KIAA1109 | -1.823 | 0.0333195 |
| LOC338799 | 1.562 | 0.0333195 |
| ABCA1 | -1.319 | 0.03332273 |
| ISG20 | -1.569 | 0.03332273 |
| PAX5 | -0.900 | 0.03343135 |
| ITGA3 | 1.728 | 0.03344922 |
| DPP4 | -1.905 | 0.03344922 |
| C21orf2 | 1.571 | 0.03373612 |
| FGF7 | -1.107 | 0.0338416 |
| LRRC59 | -1.500 | 0.0338416 |
| ZMYM2 | -1.486 | 0.03388289 |
| CFP | -1.496 | 0.03393642 |
| SERPINB8 | -1.191 | 0.03396024 |
| PRSS16 | -1.850 | 0.03410418 |
| C20orf52 | -1.283 | 0.03420829 |
| ADAMTS17 | -0.870 | 0.03451347 |
| ARMCX6 | 1.151 | 0.03455681 |
| HLF | -1.519 | 0.03463296 |
| C19orf28 | 2.268 | 0.03464471 |
| COPS4 | -1.132 | 0.03464471 |
| LRRK2 | 1.897 | 0.03465312 |
| USP15 | -1.333 | 0.03465312 |
| HDAC4 | -0.931 | 0.03477509 |
| AVPI1 | -0.871 | 0.03481799 |
| KRT3 | 1.340 | 0.03483427 |
| RPP25 | -1.674 | 0.03497029 |
| MFAP5 | -1.489 | 0.03505741 |
| TSPAN19 | -0.985 | 0.03514458 |
| FBXL3 | -1.682 | 0.03522306 |
| MANEA | -1.181 | 0.03544692 |
| NR5A1 | -0.977 | 0.03544692 |
| CEP70 | -1.232 | 0.03568662 |
| BTBD14B | 2.628 | 0.03574346 |
| VIT | 1.794 | 0.0358542 |
| CASP4 | -1.347 | 0.03592119 |
| INHA | -1.594 | 0.03592119 |
| MAL2 | -0.736 | 0.03611998 |
| LRRC25 | -0.973 | 0.03628548 |
| PRKACA | -0.912 | 0.03628548 |
| MTHFD1L | -1.310 | 0.03657509 |
| CHCHD6 | -1.094 | 0.03699808 |
| PAK3 | -1.156 | 0.03703835 |
| SMOC2 | -1.840 | 0.03703835 |
| SPRR2D | -1.884 | 0.03703835 |
| TMEM30A | -1.376 | 0.03728167 |
| HIP1 | 1.415 | 0.03737062 |
| LIPE | 1.486 | 0.03737062 |
| NKX6-2 | -1.569 | 0.03737062 |
| UQCRQ | -1.380 | 0.03737062 |
| CLDN12 | 3.481 | 0.03762749 |
| FAM38A | 3.081 | 0.03777416 |
| CNKSR1 | -1.003 | 0.03783806 |
| MVP | -0.928 | 0.0378586 |
| OR6A2 | -0.939 | 0.0378586 |
| ZNF278 | 2.313 | 0.03787617 |
| ZCCHC17 | -1.418 | 0.03809056 |
| C16orf33 | -1.813 | 0.03813932 |
| ACTRT2 | -0.960 | 0.03813932 |
| CABP7 | -0.886 | 0.03820152 |
| CRELD2 | -0.800 | 0.03820944 |
| LOC388335 | -1.197 | 0.03820944 |
| LASS5 | 1.466 | 0.03822242 |
| C6orf106 | -0.990 | 0.03825904 |
| IRF7 | -1.979 | 0.03825904 |
| PPARA | -1.164 | 0.03825904 |
| ATXN1 | 1.066 | 0.03838315 |
| NR2E1 | -1.183 | 0.03851756 |
| KCNK9 | 0.801 | 0.03856572 |
| INGX | -1.160 | 0.03856874 |
| BANK1 | 1.133 | 0.0386504 |
| DDX6 | -1.707 | 0.03896341 |
| GALNT11 | -1.029 | 0.03896341 |
| ARMCX2 | -1.091 | 0.03901828 |
| FAM102A | -1.545 | 0.03903878 |
| FEN1 | -1.445 | 0.03919467 |
| CAPN1 | -1.280 | 0.03959521 |
| FCRL5 | -1.838 | 0.03959521 |
| LRRC8C | -1.574 | 0.03959521 |
| NEK4 | 2.230 | 0.03959521 |
| SMCP | -1.519 | 0.03964551 |
| MTX2 | 1.213 | 0.03969376 |
| CRSP2 | 1.030 | 0.03987467 |
| HCP1 | -1.353 | 0.04038488 |
| PKNOX2 | -1.732 | 0.04093028 |
| SETD3 | -1.172 | 0.04112068 |
| MGC40499 | -1.154 | 0.04112922 |
| PLEC1 | 1.011 | 0.04141247 |
| ERP29 | -1.228 | 0.04152062 |
| TXNL1 | -1.357 | 0.04152062 |
| ABCC12 | -1.151 | 0.04155367 |
| TLK2 | -0.963 | 0.04161451 |
| KIAA0355 | 2.952 | 0.04184563 |
| SLC5A5 | -1.321 | 0.04234382 |
| CPNE5 | -1.481 | 0.04245244 |
| OLAH | -1.484 | 0.04297405 |
| ASB14 | 1.081 | 0.04301672 |
| SPACA4 | -1.026 | 0.04304234 |
| EIF2AK1 | -0.989 | 0.04315573 |
| CCDC104 | 1.983 | 0.04335518 |
| ANP32E | -1.776 | 0.04343195 |
| CLU | -1.195 | 0.04343195 |
| PRKRA | -0.756 | 0.04343195 |
| IER2 | 1.810 | 0.04350649 |
| P4HA1 | -1.039 | 0.04350649 |
| IFNGR2 | -2.030 | 0.04352791 |
| GALNTL2 | -1.321 | 0.044036 |
| MGC40579 | 3.679 | 0.044036 |
| H1F0 | -2.024 | 0.04417214 |
| CYB5R3 | -1.746 | 0.04437116 |
| RFX2 | -0.877 | 0.04439084 |
| ABCB9 | 0.996 | 0.04471795 |
| ITGB5 | 2.286 | 0.04487768 |
| SF3A3 | -1.279 | 0.04509844 |
| ZNF131 | -1.494 | 0.04509844 |
| THAP10 | -1.885 | 0.04519598 |
| SYNGAP1 | -0.889 | 0.04537803 |
| EIF2B3 | -1.217 | 0.04552182 |
| UBL5 | -0.936 | 0.04554348 |
| TMEM51 | 1.869 | 0.04630527 |
| ARMC2 | -0.934 | 0.04647266 |
| TRPC1 | -1.503 | 0.04660287 |
| SSX6 | -1.050 | 0.04665234 |
| CST2 | -2.094 | 0.04681638 |
| EEA1 | -0.924 | 0.04681638 |
| TSPYL2 | 0.905 | 0.04681638 |
| FTS | -0.908 | 0.04727772 |
| NDRG4 | -1.099 | 0.04733819 |
| VDAC1 | 1.282 | 0.04733819 |
| RAI16 | -1.144 | 0.0475036 |
| KLF12 | 1.349 | 0.04778336 |
| OR8B8 | 1.398 | 0.04778336 |
| SBF2 | -1.266 | 0.04778336 |
| ALAS1 | -1.384 | 0.04788023 |
| IVNS1ABP | 0.678 | 0.04809857 |
| SPOCK1 | -1.295 | 0.04841967 |
| C2orf18 | -1.574 | 0.04841967 |
| LENG8 | -1.590 | 0.04841967 |
| DDX31 | 1.130 | 0.04849452 |
| UNQ3033 | -0.937 | 0.04883509 |
| SCGB1D4 | -1.086 | 0.04902234 |
| HN1 | -1.329 | 0.04902234 |
| C6orf32 | -1.408 | 0.04902234 |
| RUVBL1 | -0.917 | 0.04902234 |
| CCDC19 | -1.516 | 0.04919888 |
| KIAA0514 | -0.887 | 0.04937679 |
| RIPK3 | -1.860 | 0.04937679 |
| CNNM2 | -0.810 | 0.04957153 |
